# Evidence showing lipotoxicity worsens outcomes in covid-19 patients and insights about the underlying mechanisms

**DOI:** 10.1016/j.isci.2022.104322

**Published:** 2022-04-27

**Authors:** Rodrigo Cartin-Ceba, Biswajit Khatua, Bara El-Kurdi, Shubham Trivedi, Sergiy Kostenko, Zaid Imam, Ryan Smith, Christine Snozek, Sarah Navina, Vijeta Sharma, Bryce McFayden, Filip Ionescu, Eugene Stolow, Sylvia Keiser, Aziz Tejani, Allison Harrington, Phillip Acosta, Saatchi Kuwelker, Juan Echavarria, Girish B. Nair, Adam Bataineh, Vijay P. Singh

**Affiliations:** 1 Division of Pulmonary Medicine and Department of Critical Care Medicine, Mayo Clinic, Phoenix, AZ, USA; 2 Department of Medicine, Mayo Clinic Arizona, Scottsdale, AZ, USA; 3 Department of Gastroenterology and Hepatology, University of Texas Health, San Antonio, TX, USA; 4 Section of Gastroenterology and Hepatology, Department of Internal Medicine, William Beaumont Hospital at Royal Oak, Royal Oak, MI, USA; 5 Mayo Clinic Alix School of Medicine, Phoenix, AZ, USA; 6 Department of Laboratory Medicine and Pathology, Mayo Clinic Arizona, Phoenix, AZ, USA; 7 Clin-Path Associates, Phoenix, AZ, USA; 8 Department of Internal Medicine, William Beaumont Hospital at Royal Oak, Royal Oak, MI, USA; 9 Department of Internal Medicine, University of Texas Health, San Antonio, TX, USA; 10 Section of Pulmonary and Critical Care Medicine, William Beaumont Hospital at Royal Oak, Royal Oak, MI, USA; 11 Department of Internal Medicine, University College of London Hospital, London, UK; 12 Division of Gastroenterology and Hepatology, Mayo Clinic Arizona, Scottsdale, AZ 85259, USA; 13 Department of Biochemistry and Molecular Biology, Mayo Clinic Arizona, Scottsdale, AZ, USA

**Keywords:** health sciences, virology, medical microbiology

## Abstract

We compared three hospitalized patient cohorts and conducted mechanistic studies to determine if lipotoxicity worsens COVID-19. Cohort-1 (n = 30) compared COVID-19 patients dismissed home to those requiring intensive-care unit (ICU) transfer. Cohort-2 (n = 116) compared critically ill ICU patients with and without COVID-19. Cohort-3 (n = 3969) studied hypoalbuminemia and hypocalcemia’s impact on COVID-19 mortality. Patients requiring ICU transfer had higher serum albumin unbound linoleic acid (LA). Unbound fatty acids and LA were elevated in ICU transfers, COVID-19 ICU patients and ICU non-survivors. COVID-19 ICU patients (cohort-2) had greater serum lipase, damage-associated molecular patterns (DAMPs), cytokines, hypocalcemia, hypoalbuminemia, organ failure and thrombotic events. Hypocalcemia and hypoalbuminemia independently associated with COVID-19 mortality in cohort-3. Experimentally, LA reacted with albumin, calcium and induced hypocalcemia, hypoalbuminemia in mice. Endothelial cells took up unbound LA, which depolarized their mitochondria. In mice, unbound LA increased DAMPs, cytokines, causing endothelial injury, organ failure and thrombosis. Therefore, excessive unbound LA in the circulation may worsen COVID-19 outcomes.

## Highlights

• Three cohorts of hospitalized COVID-19 patients with different severities were studied
• Severe COVID-19 increased serum linoleic acid (LA) and unbound fatty acid levels
• Endothelial cell uptake of unbound LA dose-dependently depolarized mitochondria
• Unbound LA increased cytokines, endothelial injury, organ failure and thrombosis

## Graphical abstract

**Figure:**
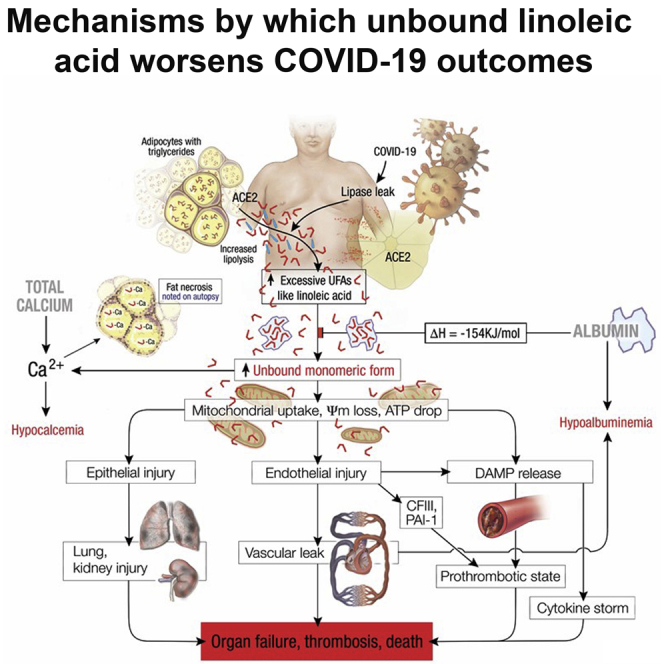

## Introduction

The coronavirus disease (COVID-19) pandemic caused by the severe acute respiratory syndrome coronavirus 2 (SARS-CoV-2) represents the greatest global public health crisis of our time. COVID-19 outcomes range from asymptomatic disease to death, with mortality being almost 50% for those requiring invasive mechanical ventilation (Domecq et al., 2021). COVID-19 outcomes therefore may depend on disease modifiers and not just infection.

SARS-CoV-2 may infect adipocytes (Martínez-Colón et al., 2021) which store triglyceride and have the angiotensin-converting enzyme 2 (ACE2) receptor (Li et al., 2020a). Rapid triglyceride breakdown by lipolysis can release large amounts of long chain (>12 carbon) non-esterified fatty acids (NEFAs) which can be toxic. Such lipotoxicity is described in severe pancreatitis, which like severe COVID-19 is worse in patients with obesity (Lighter et al., 2020; Martinez et al., 2006). In severe pancreatitis, unsaturated fatty acids (UFAs) generated by visceral triglyceride lipolysis (Camhi et al., 2011; Navina et al., 2011) comprise 60–80% of NEFAs (Navina et al., 2011; Noel et al., 2016). UFAs worsen pancreatitis by causing lung, renal and circulatory failure (de Oliveira et al., 2020b; Navina et al., 2011). Intravenous UFAs infusion is a common model of acute lung injury (Kamuf et al., 2018; Moriuchi et al., 1998). The above findings, and reports of pancreatitis or fat necrosis in 20–40% of COVID-19 full body autopsies (Hanley et al., 2020; Lax et al., 2020) suggest a potential role of lipotoxicity in worsening COVID-19.

Pancreatic lipase elevation without clinical pancreatitis correlates with worse outcomes in critical illnesses (Manjuck et al., 2005), burns (Ryan et al., 1995), trauma (Subramanian et al., 2016), hemorrhagic shock (Malinoski et al., 2009), intracranial bleeding (Justice et al., 1994), and neurosurgical intensive care unit (ICU) patients (Lee et al., 2010). The associated elevation of NEFA or their metabolites (Jeschke et al., 2004, 2008; Kosaka et al., 1994; Kreil et al., 1998; Pilitsis et al., 2002; Sztefko and Panek, 2001) supports ongoing lipolysis as being essential for the worsening. Similarly, lipase elevation is associated with worse outcomes in COVID-19 (Ahmed et al., 2021a; Goyal et al., 2021). Linoleic acid (LA), a diet derived UFA, forms 20–25% of adipose triglyceride (Guyenet and Carlson, 2015), and is very prone to lipolysis (Khatua et al., 2021). LA is elevated in patients with worse COVID-19 (Thomas et al., 2020), and dietary UFA independently associates with higher COVID-19 mortality (El-Kurdi et al., 2020). Thus, a lipolytic-lipotoxic cascade is plausible in severe COVID-19.

Most circulating NEFAs are strongly bound to albumin (El-Kurdi et al., 2020). However, a small proportion of UFAs can be unbound monomers (Richieri et al., 1993; Weisiger et al., 1981). These are biologically active because of their double bonds (Khatua et al., 2021). Unbound UFAs can rapidly induce mitochondrial toxicity (El-Kurdi et al., 2020; Khatua et al., 2021; Navina et al., 2011), and are pro-inflammatory (Khatua et al., 2021). This is supported by systemic UFA induced acute lung injury (Kamuf et al., 2018; Moriuchi et al., 1998) and multiorgan failure (MOF) (El-Kurdi et al., 2020; Khatua et al., 2021). Long chain saturated NEFAs are too hydrophobic to be monomeric in aqueous environments (Khatua et al., 2021). Unbound FAs are associated with worse outcomes in cardiac ischemia (Huber et al., 2014), perinatal hypoxia (Weinberger et al., 2001), and perhaps in severe COVID-19 (El-Kurdi et al., 2020).

Here, after initially noting UFA, total and unbound LA elevation in hospitalized patients progressing to severe COVID-19, we prospectively compared NEFAs in an ICU cohort of severe COVID-19 patients to those without COVID-19. Based on these, and the mechanistic link of UFAs triggering synchronous hypocalcemia and hypoalbuminemia (El-Kurdi et al., 2020; Khatua et al., 2020), we did a multivariate analysis to study the impact of calcium and albumin levels on mortality in a large retrospective hospitalized COVID-19 cohort. Finally, in animal experiments (mice), we administered an established LA dose (El-Kurdi et al., 2020; Khatua et al., 2021) lower than palmitic acid (PA; the most abundant saturated FA) and compared the resulting phenotype in mice to that of severe COVID-19 patients while avoiding the confounding effects of coexisting diseases (e.g., pneumonia) or therapies like fluid resuscitation. Interestingly, pre-binding LA to albumin normalized the unbound FA elevation and reversed the severe COVID-19 like phenotype induced by LA.

## Results

### Total and unbound serum oleic (OA) and linoleic acid (LA) increase in severe COVID-19 patients requiring ICU admission (cohort 1)

**Figure 1:**
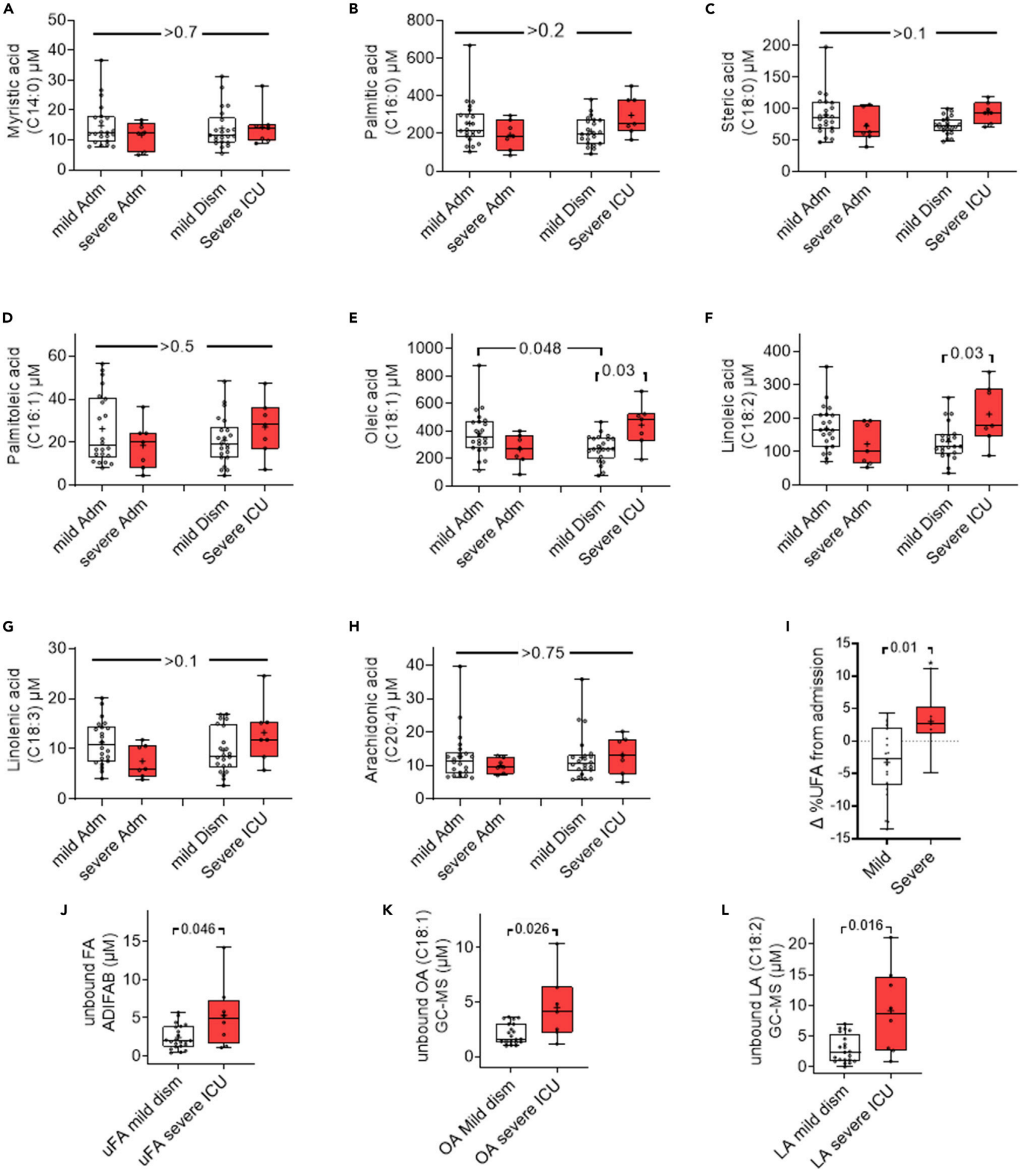
Serum fatty acid profiles (graphs) of patients hospitalized with mild and severe COVID-19 (A–L) Box plots comparing the serum fatty acid concentration in micromolars (Y-axis) at the time of admission (Adm; left pair), or dismissal to home (mild Dism) or transfer to the ICU (severe ICU) in the pair on the right. Unbound fatty acid values at the time of dismissal or ICU transfer are compared in (J–L). (J) shows unbound fatty acids measured using the ADIFAB method. (K) shows unbound oleic acid (OA; C18:1) and (L) shows unbound linoleic acid (LA; C18:2) as measured by gas chromatography-mass spectrometry (GC-MS). White boxes depict the mild cases, and red boxes the severe cases. Each point represents an individual patient. The p value shown were calculated on ANOVA with multiple comparisons (A–H) or a Mann-Whitney test for (I–L).

The COVID-19 patients dismissed home (n = 22) or transferred to the ICU (n = 8) had similar demographics (Table 1), and the interval between the first (admission) and second (dismissal or ICU transfer) blood sample (6.3 ± 2.8 versus 7.4 ± 5.9 days respectively, p = 0.51). Although NEFAs were similar at admission (Adm.,Figure 1), severe COVID-19 patients (red boxes Figure 1) had higher oleic acid (C18:1) and LA (C18:2) (Figures 1E–1F) at the time of ICU transfer. Saturated fatty acids like myristic acid (C14:0), PA (C16:0), stearic acid (C18:0), and unsaturated ones including palmitoleic (C16:1), linolenic (C18:3) and arachidonic acid (C20:4) were not significantly different between the two groups at either timepoint (Figures 1A–1D, 1G, and 1H). The other C:20 or C:22 fatty acids were also similar between the groups (data not shown). Overall, the proportion of UFAs (i.e., % UFA) at the time of ICU transfer (Figure 1I) increased in comparison to the proportion in those dismissed home. This change (Δ in Figure 1I) was measured by subtracting the % serum UFAs (of total NEFA) at the time of dismissal or ICU transfer from the respective patient’s %UFA at the time of admission. ICU transfers also had significantly higher mortality (4/8 versus 0/22, p = 0.003; bottom row of Table 1).

**Table 1:** Clinical characteristics of hospitalized COVID-19 patients, 22 of whom were dismissed home with mild disease, and 8 with severe COVID-19 transferred to the ICU

Variable	Dismissed home (22)	Transferred to ICU (8)	p value
Age in years, median (IQR)	60 (51.2–68.5)	66.5 (57.5–71.2)	0.36
Female sex, n (%)	8 (36)	1 (12)	0.37
BMI in Kg/m2, median (IQR)	30.3 (27.9–35.9)	29 (23.5–31.1)	0.25
Race, n (%)			0.27
Caucasian	16 (73)	6 (76)	
Hispanic	4 (18)	0 (0)	
African American	2 (9)	1 (12)	
Native American	0 (0)	1 (12)	
Diabetes Mellitus, n (%)	10 (45)	4 (50)	0.99
Hypertension, n (%)	11 (50)	2 (25)	0.41
Coronary artery disease, n (%)	2 (9)	3 (37)	0.1
COPD, n (%)	1 (4)	1 (12)	0.47
Cancer, n (%)	3 (14)	2 (25)	0.59
Cerebrovascular disease, n (%)	0 (0)	0 (0)	
Cirrhosis, n (%)	0 (0)	0 (0)	
Chronic kidney disease, n (%)	1 (4)	1 (12)	0.47
Immunosuppression, n (%)	3 (14)	2 (25)	0.59

**Admission Laboratory Parameters**			

White blood count (x109/L), median (IQR)	6.4 (5.1–9.2)	5.6 (4.7–8.7)	0.62
Platelets (x109/L), median (IQR)	179 (153–249)	153 (119–206)	0.32
Creatinine (mg/dL), median (IQR)	0.92 (0.76–1.04)	1.04 (0.94–1.4)	0.04
Bilirubin (mg/dL), median (IQR)	0.5 (0.3–0.7)	0.5 (0.4–0.6)	0.6
Hematocrit (%), median (IQR)	41 (39.2–44)	39.6 (36.9–43.2)	0.33
Albumin (g/dL), median (IQR)	3.9 (3.6–4.1)	3.8 (3.7–4)	0.76
Calcium (mg/dL), median (IQR)	8.7 (8.4–9.4)	8.8 (8.5–9)	0.87
C-Reactive protein (mg/L), median (IQR)	78 (55–107)	94 (63–122)	0.57

**Severity Parameters**			

SOFA score Day 1, median (IQR)	1 (1–2)	3 (2.7–3)	0.002
Mechanical ventilation, n (%)	0 (0)	4 (50)	0.003
Mortality	0(0)	4(50)	0.003

IQR: interquartile range, n: number, BMI: body mass index, ICU: intensive care unit, COPD: chronic obstructive pulmonary disease, SOFA: Sequential Organ Failure Assessment.

Because unbound FAs can worsen acute disease outcomes (Khatua et al., 2021), we compared their levels in the sera of patients at the time of dismissal to home versus ICU transfer (Figure 1J–1L) using two different methods. The fluorometric ADIFAB method, showed total unbound FAs to be statistically higher in the sera of patients transferred to the ICU (Figure 1J.). On measuring individual FAs in the de-albuminated sera by gas-chromatography and mass-spectrometry (GC-MS), we only noted serum oleic acid (OA; C18:1) and LA (C18:2) to be significantly higher in the sera of severe COVID-19 patients needing ICU care (Figure 1K and 1L). Other unbound serum saturated fatty acids (e.g., myristic acid) and unsaturated ones (e.g., palmitoleic acid) were similar between the two groups of patients (results not shown). Totals of unbound FAs measured by both methods correlated well (r = 0.73, p < 0.001), which further improved to 0.83 (CI-0.66-0.92, p < 0.001) after excluding the one definite GC-MS outlier identified using the ROUT method with a Q = 0.1%. We therefore studied if the elevated unbound LA and OA noted in ICU transfers may have worsened outcomes and resulted in mortality.

### LA and unbound FAs are elevated in ICU non-survivors, and a prospective ICU cohort of severe COVID-19 patients with hypocalcemia and hypoalbuminemia (cohort 2)

Among ICU patients, 39 had severe COVID-19 pneumonia. The non-COVID ICU patients (n = 77) were post-cardiac surgery (34, 44%), had septic shock (28, 36%) or stroke (7, 9%). Patients with COVID-19 were younger, had higher BMI, and had lesser coronary artery disease. Additional characteristics are in Table 2. COVID-19 patients received less intravenous fluids and had a more even fluid balance in the first 24 h (Table 2). Total calcium, ionized calcium, and albumin levels were significantly lower and lipase levels were significantly higher in the COVID group irrespective of septic shock in the non-COVID group (Last 4 rows Table 2, S1). Although our COVID-19 group had a higher proportion of non-Caucasian and Native-American patients, this did not affect biometrics, co-morbidities, outcomes, or measured parameters (Tables S2 and S3).

**Table 2:** Clinical characteristics of 116 patients admitted to the ICU comparing COVID versus non-COVID patients

Variable	Non-COVID (77)	COVID-19 (39)	p value
Age in years, median (IQR)	70 (56–76)	55 (44–64)	<0.0001
Female sex, n (%)	23 (30)	17 (43.6)	0.15
BMI in Kg/m^2^, median (IQR)	27.7 (24.3–32.9)	30.2 (26.8–35.8)	0.02
Race, n (%)			<0.0001
Caucasian	69 (90)	11 (28.2)	
Hispanic	5 (6)	8 (20.5)	
Asian	2 (3)	2 (5.1)	
African American	1 (1)	1 (2.6)	
Native American	0 (0)	17 (43.6)	
Diabetes Mellitus, n (%)	27 (35)	19 (48.7)	0.16
Hypertension, n (%)	33 (43)	16 (41)	0.99
Coronary artery disease, n (%)	28 (37)	5 (12.8)	0.008
Cancer, n (%)	12 (16)	2 (5)	0.13
Cerebrovascular disease, n (%)	6 (8)	3 (7.7)	0.99
Cirrhosis, n (%)	7 (9)	0 (0)	0.09
Chronic kidney disease, n (%)	14 (18)	3 (7.7)	0.17
Immunosuppression, n (%)	7 (9)	6 (15)	0.35
Pre-ICU days, median (IQR)	0 (0–1.5)	1 (1–3)	<0.0001
ICU source, n (%)			<0.0001
Emergency Room	23 (30)	3 (7.7)	
HospitalWard	11 (14)	21 (54)	
Operating Room	38 (50)	0 (0)	
Outside hospital	5 (6)	15 (38.5)	
Temperature in °Celsius, median (IQR)	37.4 (36.2–38.5)	37.5 (37–38.2)	0.34
Median arterial pressure in mmHg, median (IQR)	60 (56–64)	69 (62–77)	<0.0001
Heart rate, median (IQR)	98 (88–111)	98 (86–108)	0.55
Respiratory rate, median (IQR)	23 (19–28)	29 (24–32)	<0.0001
Fluids in first 24 h, median (IQR)	4435 (2356–5865)	2001 (1319–2638)	<0.0001
Urine output, first 24 h, median (IQR)	1735 (1325–2420)	1475 (920–2265)	0.38
Fluid balance 24 h (mL), median (IQR)	2420 (757–4047)	169 (-765-1071)	<0.0001
Glasgow Coma Scale, median (IQR)	14 (10–15)	15 (15–15)	0.0002
Acute physiologic score, median (IQR)	46 (34–58)	38 (25–62)	0.16
APACHE IV, median (IQR)	60 (45–75)	51 (34–69)	0.04
Predicted mortality, median (IQR)	8 (1.6–26)	14.7 (7.3–37.6)	0.01
Vasopressors on Day 1	52 (68)	17 (44)	0.01
SOFA score Day 1, median (IQR)	8 (5–9)	5 (4–8)	0.03
SOFA score Day 2, median (IQR)	6 (4–9)	5 (3–8)	0.33
SOFA score Day 3, median (IQR)	5.5 (3.7–9)	5 (3–8)	0.62
SOFA score Day 4, median (IQR)	4 (3–11)	6 (3–9)	0.38
SOFA score Day 5, median (IQR)	5.5 (3–10.7)	6 (4–10)	0.71
SOFA score Day 6, median (IQR)	9 (2–12)	6 (4–9)	0.75
SOFA score Day 7, median (IQR)	10 (4–12)	7 (4–10)	0.36

**Baseline laboratory results**			

White blood count (x10^9^/L), median (IQR)	11 (7.9–14.4)	9.4 (5.2–15)	0.22
Platelets (x10^9^/L), median (IQR)	144 (96–210)	226 (176–284)	<0.0001
Sodium (mmol/L), median (IQR)	139 (135–141)	136 (133–138)	0.01
Creatinine (mg/dL), median (IQR)	1.12 (0.81–1.67)	1.06 (0.81–1.68)	0.63
Blood urea nitrogen (mg/dL), median (IQR)	19 (13–27)	18 (12–35)	0.74
Glucose (mg/dL), median (IQR)	157 (133–183)	134 (107–210)	0.23
pH, median (IQR)	7.4 (7.34–7.41)	7.39 (7.31–7.44)	0.61
Lactate (mmol/L), median (IQR)	1.8 (1.3–3.9)	1.2 (1.1–1.7)	0.0003
Bilirubin (mg/dL), median (IQR)	1 (0.5–1)	0.4 (0.3–0.6)	<0.0001
Hematocrit (%), median (IQR)	29 (26–34)	38 (33–41)	<0.0001
Lipase (U/L), median (IQR)	19.5 (9–43)	73.1 (42.2–121.9)	<0.0001
Albumin (g/dL), median (IQR)	4 (3.2–4.1)	3.2 (2.9–3.4)	<0.0001
Calcium (mg/dL), median (IQR)	8.4 (7.8–9)	8 (07.4–8.3)	<0.0004
Ionized Calcium (mg/dL), median (IQR)	4.5 (4.3–4.8)	4.3 (4.1–4.5)	0.005

IQR: interquartile range, n: number, BMI: body mass index, ICU: intensive care unit, APACHE: Acute Physiology and Chronic Health Evaluation, SOFA: Sequential Organ Failure Assessment.

On serum NEFA analysis, although PA was the most abundant saturated NEFA and oleic acid was the most abundant unsaturated NEFA in both groups of ICU patients (Table 3), COVID patients had higher serum levels of LA, and percentage UFAs (Table 3). Unbound FAs were similar in the Non-COVID and COVID-19 patients (bottom row of Table 3), because of higher levels in non-COVID septic shock (5.3 μM interquartile range [IQR] 3.5–10.1μM, p < 0.001). Non-septic non-COVID controls (3.4 μM IQR 2.4–4.7μM), had lower unbound FAs than COVID-19 patients (bottom row Table S4). Serum LA, UFA, and unbound FAs were also higher in mice given LA (right side Table 3). In contrast, mice given PA (the most abundant saturated fatty NEFA) even at a higher dose than LA did not have elevated PA or unbound fatty acid levels. As shown later (Figure 7), the increase in serum LA has little significance when bound to albumin. Septic-shock patients also had elevated palmitoleic acid (21.6 μM IQR 12.2–40.4μM, p = 0.001) versus non-COVID controls (15.4 μM IQR 9.0-16.8μM). The shorter chain of palmitoleic acid with 16 carbons and 1 double bond could contribute to elevated unbound FAs (Khatua et al., 2021). Thus, septic shock, which is associated with hypocalcemia and hypoalbuminemia (Cumming, 1994), and COVID-19 both result in elevated unbound FA, albeit because of different NEFA. ICU non-survivors (n = 13) had similar demographics as survivors (Table S5). However non-survivors had higher serum LA and unbound FAs [6.2 μM IQR 4.5–11.8 μM] versus survivors [3.8 μM IQR 2.8–5.9 μM, p = 0.02 (Table S6), with a higher prevalence of MOF, ECMO requirements (Table S7) and IL-1Rα, IL-6 elevation (Table S8). Thus, elevated LA and unbound fatty acid lipotoxicity seemed to associate with worse inflammation, organ failure, and reduced survival.

**Table3:** Fatty acid profile of 116 ICU patients comparing COVID versus non-COVID patients (Left side) and experimental animal data comparing control mice versus mice administered linoleic acid (LA) or palmitic acid (PA) on the right side

Variable	Non-COVID (77)	COVID-19 (39)	p value	Control mice	LA (200mg/Kg) mice	PA (333mg/Kg) mice	p value
**Myristic acid (μM)**	8.6 (5.7–11.2)	6.7 (5.3–10)	0.11	5.9 (4.9–7.8)	6.7 (6.1–8.3)	8.7 (7.0–13.9.5)	>0.1
**Palmitic acid(μM)**	136 (114–186)	151 (116–217)	0.24	159 (148–200)	177(156–182)	142 (114–224)	>0.6
**Palmitoleic acid(μM)**	16.3 (9.6–28.6)	16.8 (9.7–24.1)	0.89	16.9 (9.1–25.9)	20.4 (18.1–22.9)	19.5 (12.9–31.5)	>0.5
**Stearic acid(μM)**	49.1 (37–58.7)	47.4 (38.4–56.1)	0.88	47.8 (38.3–57.9)	33.1 (29.5–42.0)	42.7 (35.3–49.2)	≥0.1
**Oleic acid(μM)**	226 (173–293)	243 (190–310)	0.27	75.1 (65.0–83.5)	99.0 (75.5–123)	77.0 (63.0–90.0)	>0.1
**Linoleic acid (μM)**	84.3 (60.3–110.4)	112.8 (76.2–148.5)∗	0.002	119.0 (92.7–162)	442 (229–536) ∗	131(122–212)	<0.001
**Arachidonic acid(μM)**	4 (3.2–5.3)	5.3 (3.7–6.2)∗	0.01	5.9 (4.6–6.3)	8.7 (7.8–9.1) ∗	8.3 (7.5–9.4) ∗	<0.001
**%UFA**	62.6 (60.2–64.9)	63.8 (62.5–66.3)∗	0.02	48.2 (40.1–67.6)	69.0 (59.3–73.6) ∗	43.2 (39.9–49.3)	<0.001
**Unbound FA(μM)**	3.81 (2.9–5.7)	4.75 (2.4–8.4)	0.25	5.4 (4.5–5.7)	19.4 (16.0–21.0)∗	5.4 (4.8–6.9)	<0.001

UFA: unsaturated fatty acids, FA: fatty acid. ∗ Significantly different from control group.

### Hypocalcemia and hypoalbuminemia are independent risk factors for COVID-19 hospital mortality (cohort 3)

**Figure 2:**
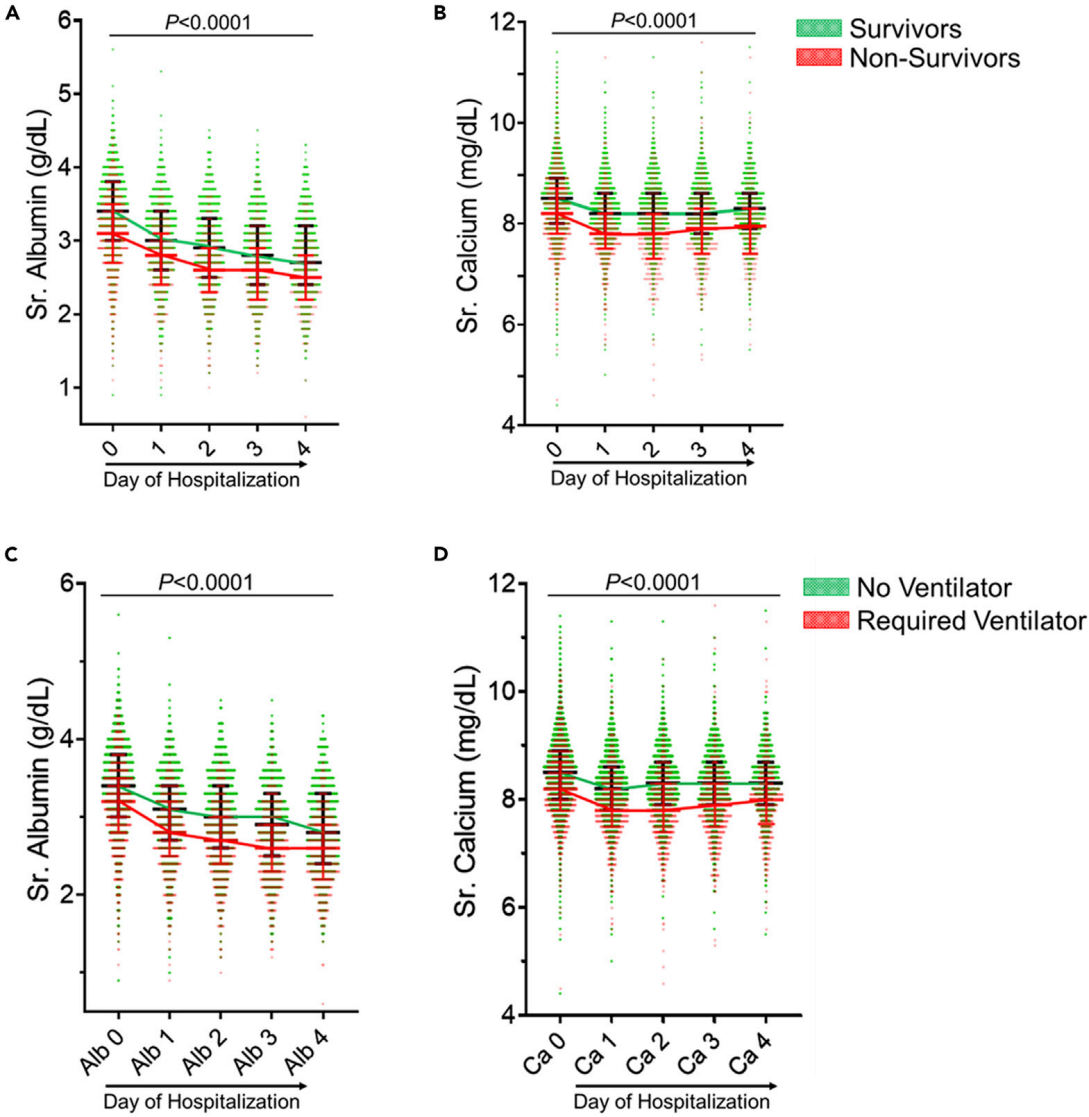
Trends of serum calcium and albumin in Cohort-3 COVID-19 patients over the first 4 days of hospitalzition (A–D) Profile of serum albumin (A and C) and calcium (B and D) in COVID-19 survivors versus nonsurvivors (A and B) or patients requiring mechanical ventilation versus not requiring mechanical ventilation (C and D). Each circle represents an individual patient. Values in all groups from day 1 to day 4 were lower than those on day 0 on ANOVA. The p value shown (p< 0.0001) was calculated on Mann-Whitney U test between survivors and non-survivors for each day separately. Patients requiring ventilator support and no ventilator are compared similarly. Survivors and patients not requiring mechanical ventilation are shown in green. The lines across connect the median values for survivors (black) and non-survivors (red). The error bars represent the interquartile range.

A total of 3969 patients were included in the retrospective multicenter study (3480 from Beaumont Health and 489 from University of Texas Health at San Antonio). Patient characteristics between hospitalized patients that survived (n = 3398) versus non-survivors (n = 571) are described in Table 4. Non-survivors presented with more comorbidities (Table 4). Calcium and albumin levels were significantly higher in survivors (as compared to non-survivors (Figures 2A and 2B) at admission and daily for the first 4 days of hospitalization (Table S9). This trend was paralleled in patients requiring mechanical ventilation (n = 781, Figures 2C and 2D). Univariate analysis showed that patients’ age, male gender, HTN, DM, CAD, CHF, CKD, history of malignancy, creatinine levels, and BUN levels had higher mortality (Table S10). On multivariate analysis, increasing patient age, BMI and male gender, unlike race or comorbidities were the only consistent variables associated with increased mortality whereas decreasing levels of calcium and albumin showed a consistent independent association with increased mortality throughout the first 4 days of hospitalization (Table 5). Individual multivariate analyses models are described in the supplemental material (Tables S11–S20).

**Table 4:** Clinical characteristics of 3969 hospitalized COVID-19 patients comparing patients that survived versus those that did not survive hospitalization

Variable	Survivors (3398)		Non-survivors (571)		p-value
	Mean	STD	Mean	STD	
Age (years)	61.56	17.39	72.66	15.06	0.001
BMI (m^2^/Kg)	32.00	8.77	30.92	9.52	0.01
CRP (mg/L) admission	117.40	88.14	125.77	85.36	0.11
Creatinine (mg/dL) admission	1.74	3.38	2.15	2.14	0.001
BUN (mg/dL) admission	24.69	22.17	37.74	26.71	0.001
Calcium (mg/dL) admission	8.48	0.69	8.26	0.87	0.001
Albumin (g/dL) admission	3.36	0.56	3.08	0.56	0.001
WBC (x10^9^/L) admission	7.79	4.06	7.86	4.46	0.71
PLT (x10^9^/L) admission	228.89	102.09	221.74	100.52	0.12
	%		%		
Male sex	49%		56%		0.001
Hypertension	51%		61%		0.001
Diabetes Mellitus	31%		36%		0.0001
CAD	11%		17%		0.0001
CKD	6%		11%		0.0001
CHF	7%		13%		0.0001
Malignancy History	8%		12%		0.0001
White Race					
Black Race					
Other Race					
Immunosuppression	2%		1%		0.35
Mechanical Ventilation	13%		65%		0.001
ICU Admission	12%		64%		0.001

STD: standard deviation; BMI: body mass index; CRP: C-reactive protein; BUN: blood urea nitrogen; WBC: white blood count; PLT: platelets; CAD: coronary artery disease; CKD: chronic kidney disease; CHF: congestive heart failure; ICU: intensive care unit.

**Table 5:** Multivariate analysis evaluating the association of hospital mortality with serum levels of calcium (A) and albumin (B)

Variable	Odds Ratio				
	Day 0	Day 1	Day 2	Day 3	Day 4
**(A) CALCIUM**					

**Age (years)**	***1.037***	***1.033***	***1.027***	***1.03***	***1.022***
**Male sex**	***1.359***	***1.294***	***1.55***	***1.451***	***1.87***
**Hypertension**	1.042	1.015	1.025	1.193	1.26
**Diabetes Mellitus**	1.115	1.178	1.179	1.071	1.107
**CAD**	1.003	0.805	0.851	0.822	0.816
**CKD**	1.146	1.139	1.313	1.126	1.332
**CHF**	1.158	1.398	1.371	1.455	1.184
**Malignancy**	1.212	1.277	1.296	1.052	1.514
**BMI (m**^**2**^**/Kg)**	***1.025***	***1.023***	***1.032***	***1.034***	***1.02***
**Creatinine (mg/dL) admission**	0.951	0.95	0.947	0.962	0.908
**BUN (mg/dL) admission**	***1.015***	***1.012***	***1.009***	1.007	***1.011***
**Black Race**	0.932	0.929	0.993	1.011	0.980
**White Race**	0.991	0.761	0.839	0.784	0.762
**Calcium (mg/dL)**	***0.678***	***0.426***	***0.373***	***0.454***	***0.45***

**(B) ALBUMIN**					

**Age (years)**	***1.036***	***1.037***	***1.04***	***1.041***	***1.039***
**Male sex**	***1.407***	***1.433***	***1.709***	***1.498***	***1.538***
**Hypertension**	1.019	0.88	1.09	1.045	1.041
**Diabetes Mellitus**	0.985	1.058	0.968	0.92	0.841
**CAD**	1.028	0.972	0.894	1.044	1.086
**CKD**	1.073	0.776	1.088	0.734	1.041
**CHF**	1.096	1.322	1.267	0.931	0.825
**Malignancy**	1.279	***1.584***	1.257	1.328	1.065
**BMI (m**^**2**^**/Kg)**	***1.028***	***1.042***	***1.041***	***1.041***	***1.034***
**Creatinine (mg/dL) admission**	0.981	1.052	0.997	1.05	1.009
**Black Race**	1.119	***1.681***	***2.073***	***1.934***	***2.087***
**White Race**	1.344	0.909	1.040	0.967	0.924
**BUN (mg/dL) admission**	***1.008***	1.004	1.003	1.005	1.001
**Albumin (g/dL)**	***0.472***	***0.424***	***0.319***	***0.376***	***0.333***

BUN: blood urea nitrogen; CAD: coronary artery disease; CKD: chronic kidney disease; CHF: congestive heart failure; BMI: body mass index. Bold italicized values have a p < 0.05.

### Animal and *in vitro* studies: LA reacts favorably with calcium and albumin, inducing hypocalcemia, hypoalbuminemia

**Figure 3:**
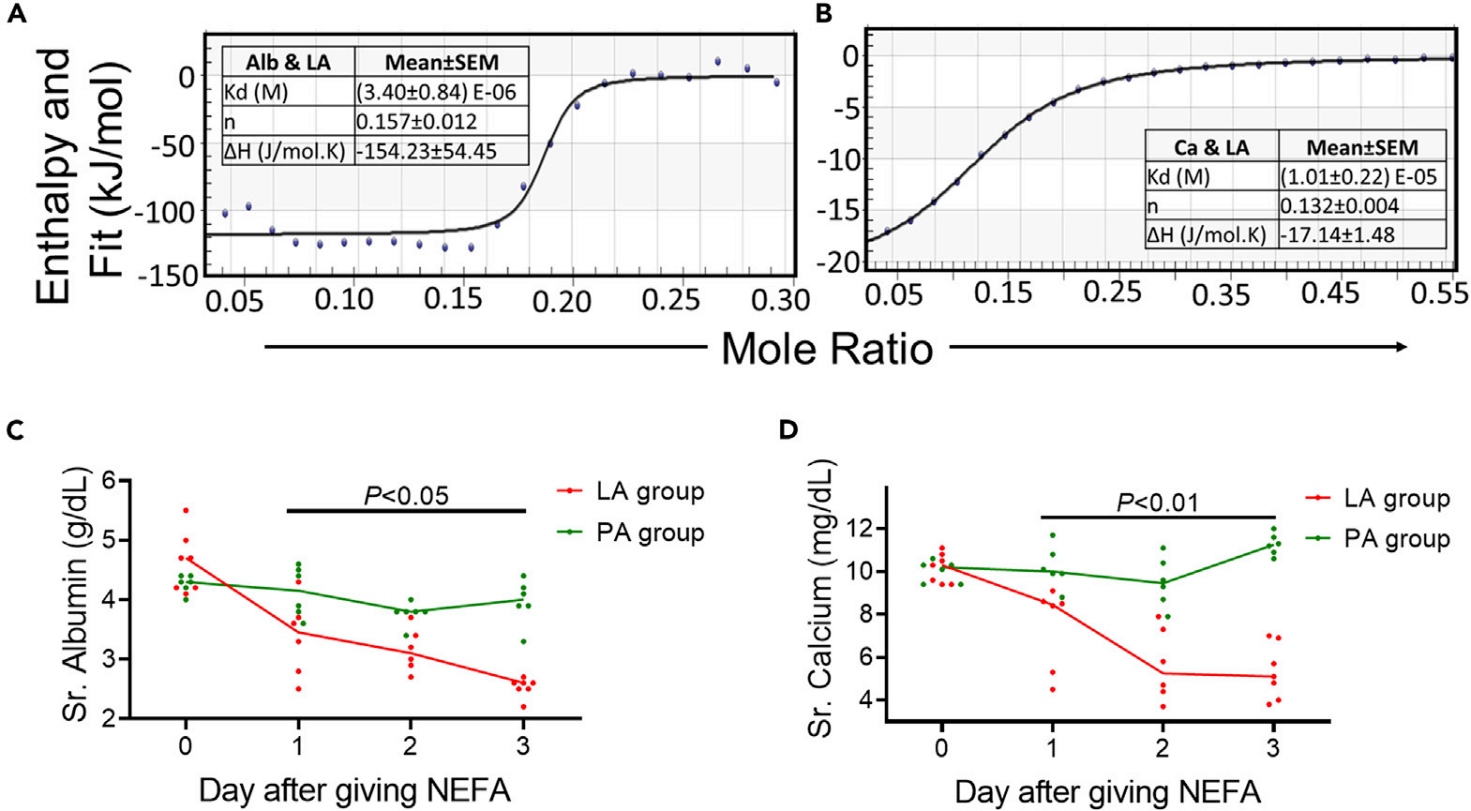
Energetics of LA’s interactions with albumin and calcium, and its effect on serum calcium and albumin in mice (A–D) Enthalpograms of injection of albumin (Alb; A) or calcium chloride (Ca; B) into LA. The various thermodynamic variables (Mean ± SEM (standard error of mean)) from 4-6 different experiments are mentioned in the Table adjacent to the enthalpogram. Time course of serum values of calcium (C) or albumin (D) in mice who were given palmitic acid (PA; green) or linoleic acid (LA; red) intraperitoneally. p values comparing the PA and LA groups for each day were calculated using a Mann-Whitney U test.

To understand hypocalcemia and hypoalbuminemia from LA elevation during COVID-19 infection, we studied their interactions using isothermal titration calorimetry. LA interacted with a favorable enthalpy (ΔH) with both albumin (ΔH = −154 ± 54 kJ/mol) and calcium (ΔH = −17.1 ± 1.5 kJ/mol) (Figures 3A and 3B). Mice given LA developed hypocalcemia and hypoalbuminemia (Figures 3C and 3D) over a few days as in patients with COVID-19 associated mortality (Figure 2). We thus went on to study if the increased unbound FAs noted in COVID-19 patients and mice with elevated LA cause MOF, thrombosis, and cytokine elevation noted in severe COVID-19.

### Severe COVID-19 and unbound LA cause endothelial damage

**Figure 4:**
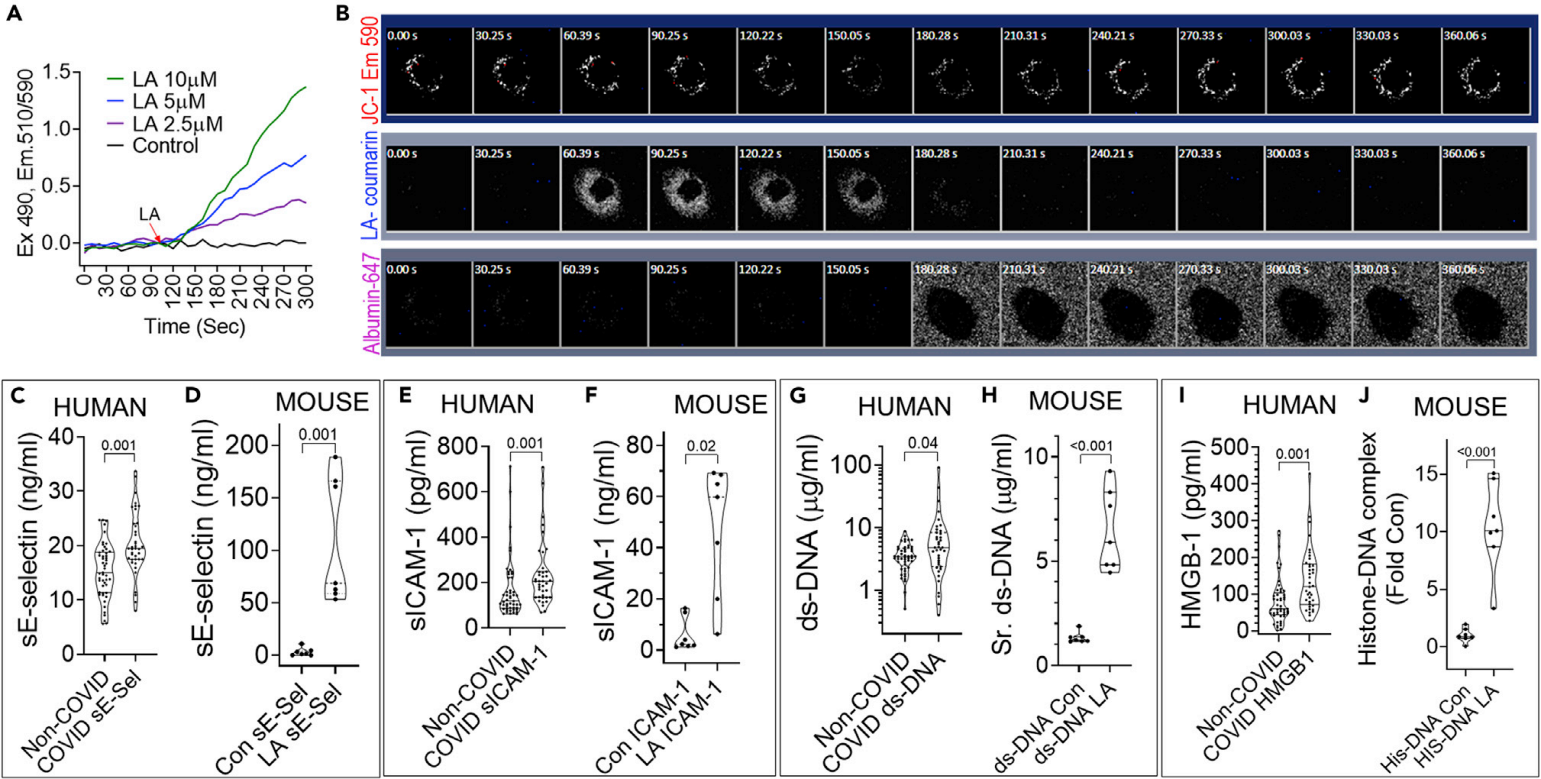
Comparison of COVID-19 induced endothelial damage and DAMP release to that induced by LA, and mechanisms that underlie this damage (A) Representative experiment showing the time course of LA induced mitochondrial depolarization as measured in a HUV-EC cell suspension loaded with the mitochondrial membrane potential sensitive dye JC-1 in a stirred fluorometer cuvette. The Emission (Em) 510/590 ratio after excitation (Ex) at 490 nm (Y-axis) depicts the loss of mitochondrial membrane potential. The different concentrations of LA (color coded) were added at 100 s. The black line shows untreated cells. (B) Image time series (time in seconds on top left of each image) of adherent Mito-tracker red (MT-Red) loaded HUV-ECs showing the time course of loss of red fluorescence (top row) on uptake of 30 μM LA, with 1.5 μM of LA-coumarin tracer(blue) added at 60.39 s (LA-coumarin, middle row). Fluorescently tagged albumin (Albumin-647; bottom row) was added at 180 s. Note albumin extracts the LA from the cells, restoring the MT-Red fluorescence. (C–J) Violin plots showing serum levels of soluble E-selectin (C and D), soluble ICAM-1 (E and F), ds-DNA (G and H), HMGB-1 (I) and Histone-DNA complexes (J) in Non-COVID, non-septic shock patients versus COVID positive ICU patients at admission (C, E, G, and I) or control (Con) mice versus mice which had received LA 24 h before (D, F, H, and J). Each dot represents an individual patient or mouse. The p-values shown above were calculated using a Mann-Whitney test. p values comparing the two groups of humans, or two groups of mice were calculated using a Mann-Whitney U test.

**Figure 5:**
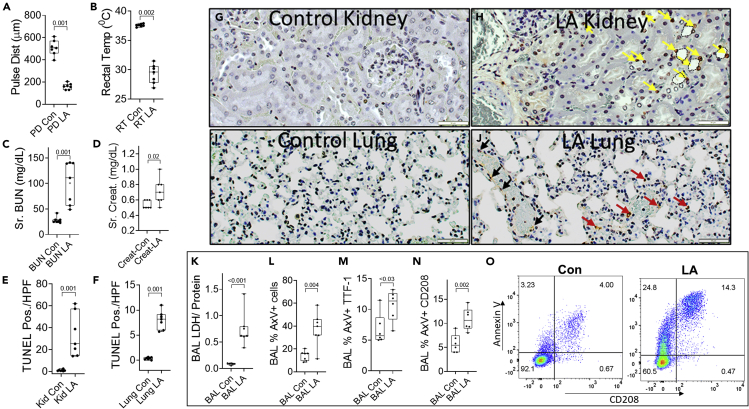
Organ failure induced by LA in mice (A–F) Box plots of control (Con) or LA treated mice comparing carotid artery pulse distention (Pulse Dist (PD); A) as a measure of shock, Rectal temperature (RT; B) as a measure of severe inflammatory response syndrome, blood urea nitrogen (BUN; C), creatinine (Creat.; D) as a measure of renal failure. TUNEL positive cells/ high power field in the renal cortex (E), and lung (F) as measures of cell death in these organs. (G–J) The images are representative TUNEL stained histologic sections, showing brown TUNEL positive cells. In the kidney panels (G and H), the yellow arrows show some of the TUNEL positive nuclei in renal tubules, and the dashed outlines show some examples of the dilated lumina of tubules, with loss of brush border. In the lung panels (I and J), black arrows denote the TUNEL positive cells in the blood vessels and red arrows denote the positive cells in the alveoli. (K–N) (K) Ratio of lactate dehydrogenase (LDH) activity by protein content in the bronchoalveolar lavage (BAL) fluid collected at the time of necropsy. L-N show annexin V positive (AxV+) staining in all BAL cells (L), Thyroid transcription factor-1 (TTF-1) positive (M), or CD208 positive cells (N). (O) shows representative flow cytometry scattergrams of BAL cells stained for CD208 and annexin V. p values calculated by a Mann-Whitney U test are shown above corresponding box plots.

We first noted that unbound LA at clinically relevant concentration range of 2.5–10 μM dose dependently depolarized mitochondria (510/590 Emission ratio in Figure 4A) in JC-1 loaded endothelial cells (HUV-EC). The phenomenon seemed to be triggered by unbound LA since LA (30 μM) and its fluorescent tracer LA-coumarin were reversibly taken into HUV-ECs (middle row 60-150S images, Figure 4B). Although LA uptake depolarized HUV-EC mitochondria as evidenced by reduced fluorescence of the mitochondrial membrane potential sensitive dye MitoTracker Red CMXRos (MT-Red, top row), the addition of albumin and a fluorophore (Albumin-647, at 180s, bottom row) reversed the uptake and depolarization. Thus, unbound LA may depolarize endothelial mitochondria and injure them. This hypothesis is supported by the higher endothelial injury marker; soluble E-selectin in ICU COVID-19 patients (versus non-septic COVID-19 controls), and LA treated mice (Figure 4C and 4D). In later sections (Figure 5J, black arrows) we also noted morphological evidence of LA induced endothelial injury *in vivo*. Similar to COVID-19 patients, LA treated mice had higher soluble ICAM-1, and DAMPs (i.e., ds-DNA, HMGB-1, and histone-DNA complex levels) (Figures 4E–4J) which as we shall discuss later can be pro-thrombotic and pro-inflammatory.

### Severe COVID-19 induced cytokine elevation, organ failure and thrombosis in ICU patients are replicated by unbound LA *in vivo*

COVID-19 ICU patients had significantly higher levels of the proinflammatory cytokines CXCL1, IL-1β, IL-6, MCP-1, and TNF-α compared to the non-COVID group (Table 6). LA treated mice also had a similar pattern of cytokine elevation versus controls (right side Tables 6, S21). However, in PA administered mice, although IL-6 elevation was noted, it was lower than in mice given LA (right side Table 6).

**Table 6:** Cytokine profile of 116 ICU patients comparing COVID versus non-COVID patients (left side) as well as animal experiment comparing control mice versus mice treated with linoleic acid (LA) on the right side

Variable	Non-COVID (77)	COVID-19 (39)	p value	Control mice	LA (200 mg/kg) mice	PA (333mg/Kg) mice	p value
**GROα (pg/mL)**	19.5 (7.3–33.5)	35 (18.6–53.8)	0.001	685 (480–1018)	65,034 (63,915–67,163)^a^	760 (350–1493)	0.001
**IL-1β (pg/mL)**	2.75 (0–17.75)	8.3 (4.6–16.5)	0.04	8.5 (1.2–19.0)	26.8 (15.8–46.8)^a^	3.7 (1.2–7.4)	0.01
**IL-1Rα (pg/mL)**	8 (3.1–72.9)	22.4 (9.1–53.1)	0.01				
**IL-4 (pg/mL)**	2.32 (0–6.5)	4.7 (1.4–6.9)	0.03	5.0 (0.6–8.1)	7.5 (5.6–7.5)	2.4 (0.1–2.7)	0.34
**IL-6 (pg/mL)**	146.8 (34.8–422.3)	360 (67–641)	0.02	22.8 (3.1–155)	5828 (4383–11,758)^a^	531 (446–837)^b^	0.001
**IL-18 (pg/mL)**	50.8 (29.7–114)	119.4 (86.4–174.2)	0.01				
**IP-10 (pg/mL)**	214 (120–556)	3204 (1956–3941)	<0.001	576 (446–783)	926 (573–1318)	380 (296–480)	0.23
**MCP-1 (pg/mL)**	578 (373–858)	1336 (892–1836)	<0.001	126 (59–339)	2350 (1993–2526)^a^	271 (217–279)	<0.001
**TNFα (pg/mL)**	17.6 (6.5–49.9)	40.4 (28.3–58.4)	<0.001	25.0 (13.7–43.5)	120 (108–127)^a^	29.1 (10.2–31.7)	<0.001

a Significantly different from control and Palmitic acid (PA) treated mice.

b Significantly different from control mice.

**Figure 6:**
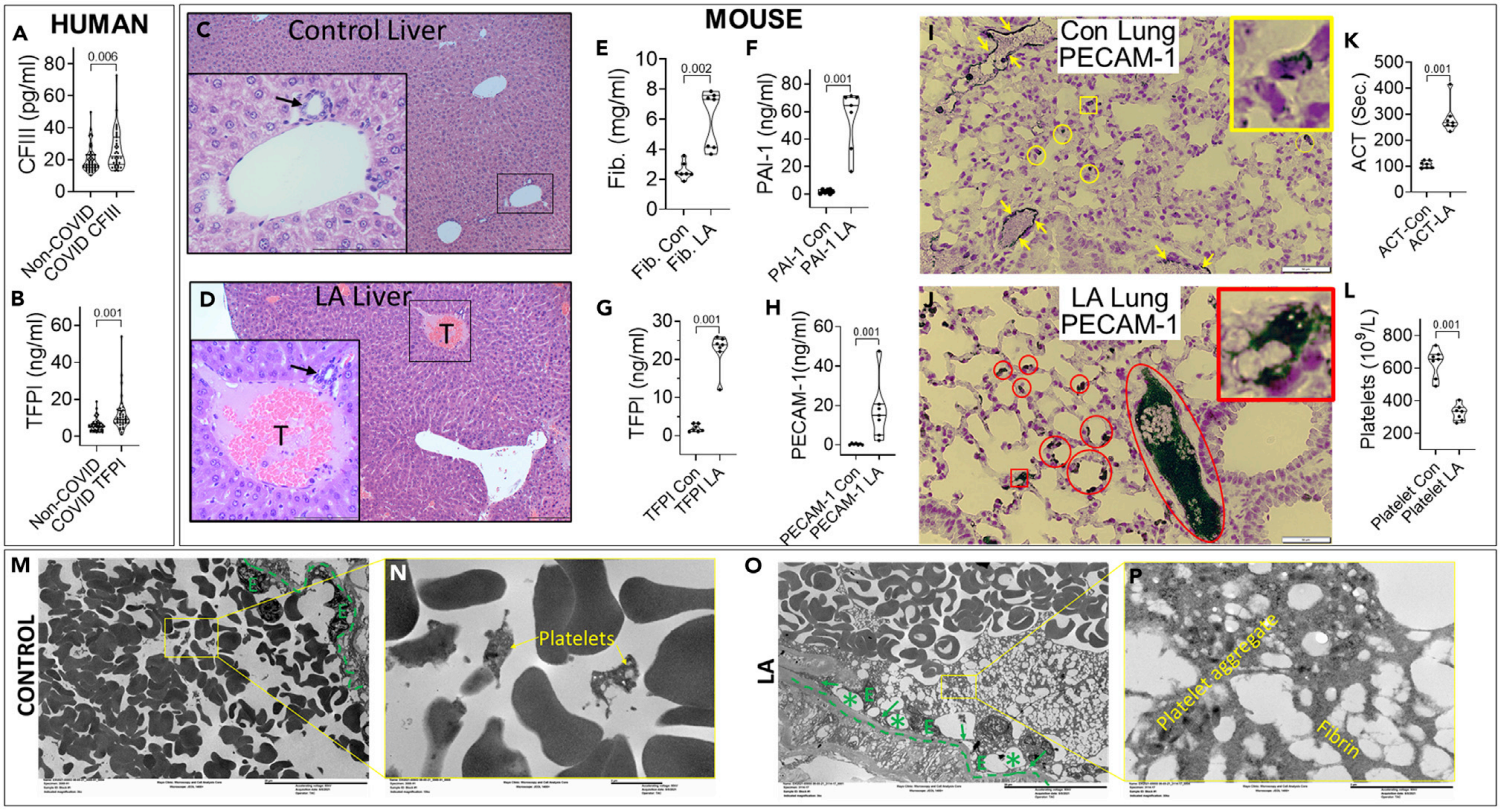
Comparison of parameters for thrombosis in COVID-19 patients and mice given LA (A–I) Violin plots showing coagulation factor III (CFIII; A) and tissue factor pathway inhibitor (TFPI; B) in the sera of patients admitted to the ICU for COVID and non-COVID, non-septic indications. Hematoxylin and erosion-stained liver sections in control mouse (C) and LA treated mouse (D) livers. The insets and magnified images show a portal venule with an adjacent biliary ductule (black arrow) in both C and D. Note the thrombus (T) in the portal venule of the LA treated mouse. Mouse plasma fibrinogen (Fib.; E), plasminogen activation inhibitor-1 (PAI-1; F), TFPI (G) and PECAM-1 (H) in controls (Con) and those after LA treatment. Immunohistochemistry of PECAM-1 in the lungs of control mice (I), with yellow arrows pointing to the positive PECAM-1 staining in normal pulmonary vascular endothelium. Note a few white cells in the vessel also stain positive. The yellow circles and square (with large inset) show a few alveolar capillaries that stain positive for PECAM-1. (J–P) (J) Immunohistochemistry of PECAM-1 in the lung of an LA treated mouse. The red circles and square (with large inset) highlight the strongly positive staining in several alveoli and capillaries. The large oval shows strong PECAM-1 staining in a large thrombus in a pulmonary vessel. Note platelets are positive for PECAM-1. (K) Activated clotting time (ACT) and platelet count (L) in the blood of control and LA treated mice. P values calculated by a Mann-Whitney U test are shown above corresponding violin plots. Electron microscopic images of pulmonary vessels from control (M and N) or LA treated (O and P) mice. M and O reveal the low power images, and N, P reveal magnified views of the rectangles shown in M, N. The dashed green line shows the basement membrane, and endothelial cells are shown as “E”. The green arrows in O reveal areas where fibrin strands contact the basement membrane and ∗ reveal areas where the endothelial cells are lifted off or absent from the basement membrane. The scale bars (200 μm for C, D and 50 μm for I, J and 20 μm for M, O and 2 μm for N and P) are shown to the bottom right corner of each image.

COVID-19 patients were in a prothrombotic state evidenced by a higher rate of deep venous thrombosis (DVT) and pulmonary embolism (PE), irrespective of septic shock (Bottom rows of Tables 7, S22), whereas MOF was higher after excluding septic shock (Top rows of Tables 7, S22). ECMO utilization was also seen more frequently in the COVID-19 group (Tables 7, S22). Similar to COVID-19 patients, LA induced MOF in mice (Figure 5) necessitating euthanasia by 72 h. Shock was evidenced by a drop in carotid artery pulse distention (Pulse dist., Figure 5A). LA also caused hypothermia (Figure 5B) suggesting a severe systemic inflammatory response. Apart from shock, LA also induced other parts of MOF. LA induced renal failure was noted as a large increase in serum BUN and creatinine (Figures 5C and 5D). LA also increased TUNEL (Terminal deoxynucleotidyl transferase dUTP nick end labeling) positivity in the renal tubules (Figures 5E and 5H yellow arrows). LA induced lung injury was noted as TUNEL positivity in the lung alveoli (Figures 5F and 5J red arrows) and vessels (J, black arrows). Interestingly, this was associated with pulmonary thrombi (Figures 5J and 6J). LA induced lung injury was also noted as increased lactate dehydrogenase (LDH) and higher annexin V positive cells in the bronchoalveolar lavage (BAL; Figures 5K–5L). Specifically, type-II pneumocyte injury was noted as increased dual Thyroid transcription factor-1 (TTF-1), Annexin V positive cells, or CD208 + Annexin V+ cells (Figures 5M−5O) in the BAL of LA treated mice. Therefore, LA induced shock, and renal and lung injury in mice.

**Table 7:** Outcomes and interventions of 116 patients admitted to the ICU comparing COVID versus non-COVID patients

Variable	Non-COVID (77)	COVID-19 (39)	p value
Multiorgan failure, n (%)	32 (42)	23 (59)	0.08
Renal Replacement Therapy, n (%)	8 (10)	9 (23)	0.09
Veno-venous ECMO, n (%)	2 (3)	10 (26)	0.0003
ICU Mortality, n (%)	7 (9)	5 (12.8)	0.53
Mechanical ventilation days, median (IQR)	0.5 (0.3–1.8)	25.8 (9.4–44.9)	<0.0001
ICU length of stay days, median (IQR)	2.2 (1.4–3.8)	14.5 (5.6–34.6)	<0.0001
Hospital length of stay days, median (IQR)	7.1 (5.1–11.1)	22.9 (10.7–42.2)	<0.0001
28 Day Mortality, n (%)	8 (10)	5 (12.8)	0.75
DVT or PE events, n (%)	3 (4)	10 (26)	0.0009

ICU: intensive care unit, IQR: interquartile range, ECMO: extracorporeal membrane oxygenation, DVT: deep venous thrombosis, PE: pulmonary embolism.

On focusing on prothrombotic mechanisms, we noted COVID-19 patients in the ICU had higher coagulation factor III (CFIII; Figure 6A) and tissue factor pathway inhibitor (TFPI; Figure 6B) supporting a prothrombotic state compared to controls. Similarly, 8 of 12 mice given LA had portal venous thrombi (Figures 6C and 6D) unlike control mice. A prothrombotic state induced by LA was noted as increased fibrinogen, plasminogen activation inhibitor-1 (PAI-1), and TFPI levels. Elevated Soluble Platelet Endothelial Cell Adhesion Molecule 1 (PECAM-1) or CD31 (Figures 6E-H) levels suggested LA induced endothelial injury *in vivo*. On immunohistochemistry of control mouse lungs, PECAM-1/CD31 normally localized to pulmonary vessel endothelium (yellow Figure 6I), and immune cells as described previously (Lertkiatmongkol et al., 2016). LA, however, dramatically increased PECAM-1/CD31 expression in the alveolar capillaries and pulmonary vascular thrombi (red squares, oval, Figure 6J), with the mice developing thrombocytopenia and an elevated activated clotting time (Figures 6K–6L). Electron microscopy of pulmonary vessels showed LA causes loss of or lifting of endothelial cells (∗ in Figure 6O) from the basement membrane, wherein fibrin strands attached (green arrows Figure 6O) and extended inwards towards large platelet aggregates (Figure 6P).

**Figure 7:**
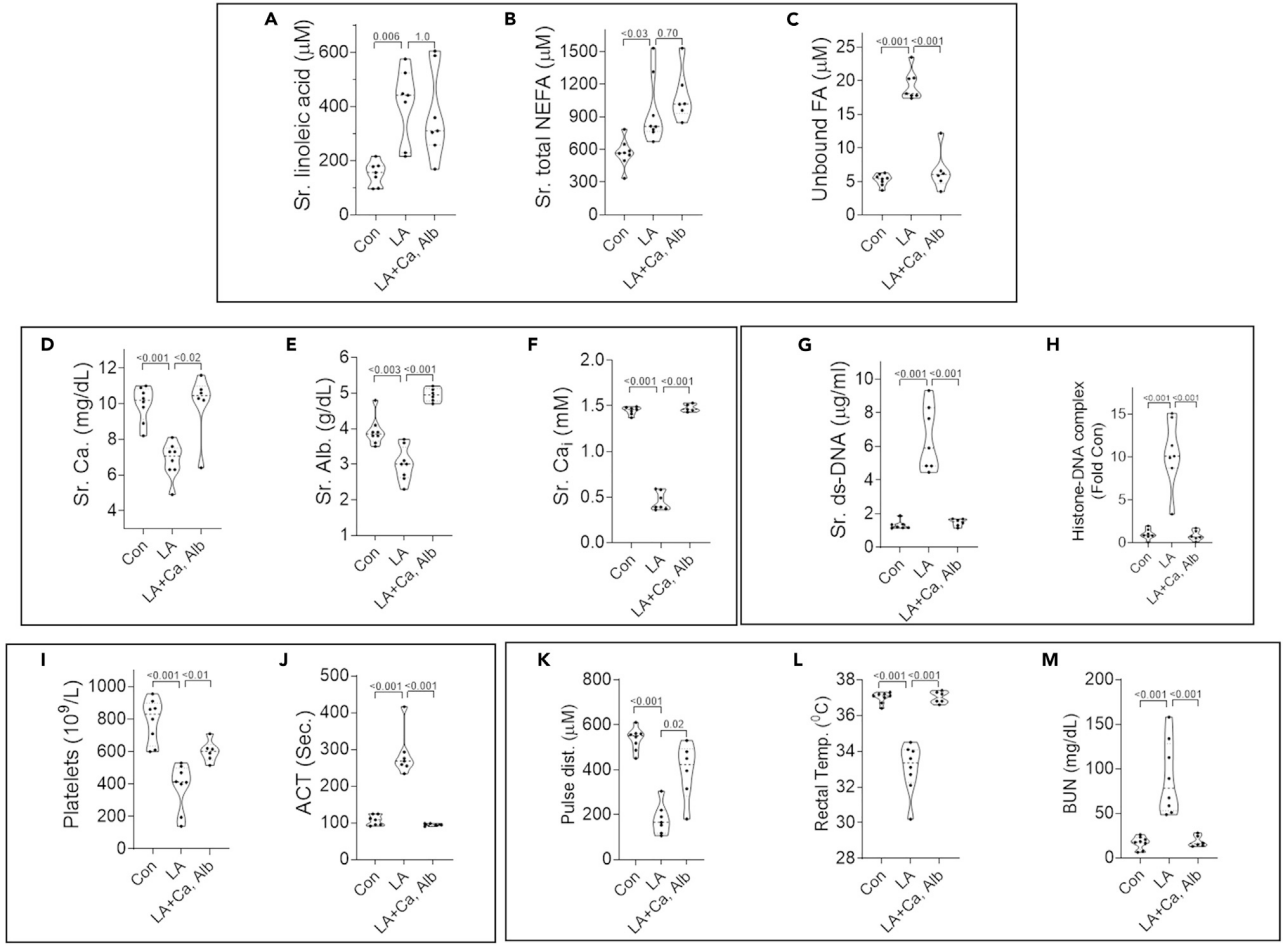
Impact of calcium, albumin co-administration on LA induced changes in fatty acids, calcium, albumin, DAMPs, coagulation abnormalities, and multisystem organ failure (A–L) Violin plots of control mice (Con), 48 h after LA administration (LA), and 48 h after administration of LA incubated with albumin and calcium (LA + Ca,Alb). Shown are serum linoleic acid measured by GC-MS (A), total non-esterified fatty acids measured using the colorimetric LabAssay ™ NEFA kit (NEFA; B), unbound fatty acids (C), total calcium (Ca; D), Albumin (Alb.; E), ionized calcium (Ca_i_; F), The DAMPs shown are double stranded DNA (ds-DNA; G), and histone-DNA complexes (H). Effects on coagulation parameters including platelets (I) and activated clotting time (ACT; J) are also shown. Carotid pulse distention (Pulse dist. K); a measure of shock, rectal temperature (L); a measure of severe inflammatory response, and blood urea nitrogen (BUN, M) are also shown. P values calculated by a Mann-Whitney U test are shown above corresponding violin plots.

Administration of LA with calcium and albumin (fatty acid free) inhibited the increase in unbound FA without affecting the increase in serum LA or total NEFA (Figures 7A–7C). This reduction in unbound FA normalized serum calcium, albumin, and ionized calcium (Figures 7D–7F) prevented DAMP increase (Figures 7G–7H), coagulation abnormalities (Figures 7I–7J) and MOF (Figures 7K–7M) although significantly (p< 0.001) improving survival at 72 h (0/7 with LA versus 6/6 with LA + Ca, Alb). Thus, in mice unbound LA may mediate most of the severe COVID-19 like outcomes noted in humans.

Finally, it has been reported that patients with diabetes mellitus could be linked to underlying elevated FAs as compared to non-diabetics (Bergman and Ader, 2000). Furthermore, LA plasma levels have been reported to be significantly higher in women (Lohner et al., 2013). In order to evaluate if the presence of diabetes or the female gender could confound the results, we analyzed the ICU cohort (Cohort 2) firstly by removing all diabetic patients (Table S23) and secondly by removing women from the cohort (Table S24). No major differences were noted as compared to the initial results. Therefore, the prothrombotic state, MOF and reduced survival noted in severe COVID-19 patients is likely because of elevated unbound UFAs like LA.

## Discussion

Here we present evidence in humans with mechanisms in animals and *in vitro* studies explaining how unbound LA may worsen COVID-19 outcomes. Prospectively, in hospitalized patients we note that in comparison to mild COVID-19 patients, those progressing to severe COVID-19 requiring ICU admission have higher UFAs, including LA and OA, which are also increased in the unbound form. Severe COVID-19 ICU patients had higher lipase, LA levels, UFAs, inflammatory cytokines, thrombotic events, hypoalbuminemia, and hypocalcemia compared with non-COVID patients. Unbound FAs were elevated in both COVID-19 and septic shock patients who also had a similar rate of MOF. Cumulatively, COVID-19 patients required more organ support therapies including ECMO and mechanical ventilation. The retrospective hospitalized COVID-19 cohort had hypocalcemia and hypoalbuminemia independently associated with hospital mortality and ventilator requirements after adjusting for age, gender, BMI, race, and medical comorbidities.

Experimentally in mice, unbound LA induced the widely reported hypoalbuminemia, hypocalcemia, DAMP release, cytokine storm, thrombosis, and MOF phenotype, which we note and others have reported in severe COVID-19. These are consistent with well-known models of acute lung injury induced by intravenous UFAs (Kamuf et al., 2018; Moriuchi et al., 1998). PA, the most abundant saturated NEFA, despite being administered at higher doses than LA, did not enter the circulation, perhaps because of its extreme hydrophobicity (Khatua et al., 2021). Administering intraperitoneal PA with a solvent (dimethyl sulfoxide) or directly through an incision were equally harmless (data not shown).

**Figure 8:**
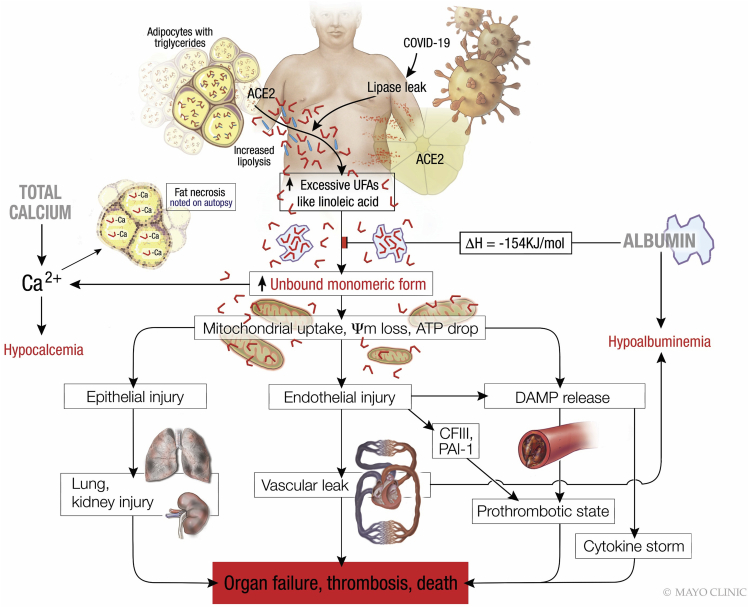
Schematic describing the pathophysiology observed in severe COVID-19, wherein lipolytically generated LA results in the organ failure, thrombosis and death preceded by hypocalcemia and hypoalbuminemia The red L shaped structures denote unsaturated fatty acids like LA, which are present in excess although the blue straight lines denote saturated fatty acids. When the amount of unsaturated fatty acids like LA exceeds the ability of albumin to bind them, the unbound LA reacts with calcium causing hypocalcemia. Excess unbound LA is taken up by cells, causing mitochondrial depolarization, and consequent epithelial and endothelial injury with DAMP release, the latter among which causes the cytokine storm. The endothelial injury causes vascular leak, and hypoalbuminemia in addition to the release of procoagulant coagulation factor III (CFIII) from basement membranes, which with DAMPs promote thrombosis. These plus plasminogen activation inhibitor-1 (PAI-1) result in a prothrombotic state and worsening organ failure which can result in death.

Although albumin binds LA more strongly than calcium (ΔH = −154 ± 54 kJ/mol versus −17.1 ± 1.5 kJ/mol; Figures 3A and 3B), albumin’s molar amounts in normal serum (600–800 μM) are lower than calcium (2–2.5mM). However, albumin’s stronger binding to LA put it upstream of calcium in preventing lipotoxicity (Figure 8). Therefore, despite the increase in total LA concentrations induced by giving prebound LA (Figure 7B); the prebinding kept unbound FAs low at control mouse levels (Figure 7C). This is mechanistically consistent with the energetically favorable pre-binding of LA by albumin preventing the increase in unbound FAs despite an increase in total LA. Although we cannot comment on the exact proportion of unbound LA neutralized by calcium, the therapeutic role of calcium is supported by it preventing LA induced hypocalcemia (Figures 7D and 7F) and its energetically favorable binding to LA (Figure 3B). This is clinically relevant because we note hypocalcemia with severe COVID-19 (Figure 2), and calcific fat necrosis is noted in autopsies of COVID-19 patients (Lax et al., 2020). The protective role of calcium against lipotoxicity is also supported by previous studies showing extracellular calcium deposits in fat necrosis and extracellular calcium supplementation to reduce lipotoxic cell injury and delay organ failure (Khatua et al., 2020). Mechanistically, unbound LA relevant to COVID-19 concentrations was reversibly taken into cells and depolarized mitochondria (Figures 4A and 4B). LA which reduces transendothelial resistance, causing macromolecular capillary leakage (El-Kurdi et al., 2020; Khatua et al., 2021), reacted with albumin and calcium, explaining the rapid hypoalbuminemia and hypocalcemia we note in humans and mice (Figures 2 and 3). Such hypoalbuminemia cannot be explained by reduced albumin synthesis, because albumin has a 25-day half-life (Levitt and Levitt, 2016). These findings and concepts are summarized in Figure 8.

Although most COVID-19 infections are mild or self-limited, in this study a severe phenotype-like picture could be induced by an UFA increased in the blood of patients with severe COVID-19, i.e., LA. This along with the use of intravenous UFAs to induce lung injury (Kamuf et al., 2018; Moriuchi et al., 1998) supports LA’s role in worsening COVID-19. Our ICU COVID-19 patients, as in previous studies (Domecq et al., 2021; Richardson et al., 2020) developed respiratory failure (Domecq et al., 2021; Li et al., 2021), MOF, and VTE events. Similarly, endothelial injury (Ackermann et al., 2020), lung injury, and vascular occlusions were also induced by unbound LA in mice (Figures 5 and 6). LA induced injury in the BAL without coexisting pneumonia or pancreatitis suggest unbound LA may worsen COVID-19. Interestingly, the elevation of unbound FAs, UFAs, and LA in ICU non-survivors (Table S6) irrespective of etiology suggests a broader relevance of such elevations. Please note that because our COVID-19 patients had an even fluid balance, we gave our mice water *ad libitum* with subcutaneous saline supplementation at 10% bodyweight/ day.

Covalently bound LA is not lipotoxic and is present in intravenous triglyceride lipid formulations(Ahmed et al., 2021b), propofol, and as several kilograms of adipose triglyceride(Guyenet and Carlson, 2015).The role of lipolysis dependent lipotoxicity in worsening COVID-19 is supported by: 1) presence of ACE2 receptor in pancreas and fat (Ackermann et al., 2020); 2) SARS-CoV-2 infecting adipocytes (Martínez-Colón et al., 2021); 3) meta-analysis of 21 studies with 34,496 COVID-19 patients showing elevated lipase associates with increased mortality (Yang et al., 2022), which is also noted for obesity (Lighter et al., 2020); 4) higher serum LA in COVID-19 patients with elevated IL-6 (Thomas et al., 2020); 5) presence of pancreatitis, and fat necrosis (Hanley et al., 2020; Lax et al., 2020) reported in whole body COVID-19 autopsies; and 6) both severe pancreatitis (Hegyi et al., 2020) and COVID-19 having cytokine elevation like LA.

The proportion of LA in human adipose increased from 5 to 10% in the 1950s to ≥20% by 2000 (Guyenet and Carlson, 2015), closely following the pattern of increased dietary intake of LA(Blasbalg et al., 2011). Although LA (50 μM) was proposed to synergize with the anti-viral drug Remdesivir and reduce SARS-CoV-2 replication in human cells(Toelzer et al., 2020); the reduced replication can be explained by LA’s toxicity on the cells in which the virus was cultured. This diet related increase in visceral LA and association of COVID-19 mortality with UFA intake noted on multivariate analysis (El-Kurdi et al., 2020) along with the inability of remdesivir to reduce COVID-19 severity (Al-Abdouh et al., 2021; Okoli et al., 2021; Piscoya et al., 2020) suggest a deleterious role of excess LA in COVID-19 infection.

Double bonds in a FA, like LA, increase its lipolytic generation, and the aqueous stability of its monomers even without a carrier like albumin (Khatua et al., 2021). Saturation in contrast makes long chain fatty acids too hydrophobic to exist as unbound monomers. This likely explains the lack of PA elevation, cytokine response in PA administered mice (Tables 3 and 6) and the lack of MOF as previously reported (Khatua et al., 2021). Double bonds explain the elevated UFAs and unbound FA levels in COVID-19 non-survivors (Table S6), those with septic-shock [who had increased palmitoleic acid (C16:1), the shorter chain of which increases aqueous stability] and mice given LA alone (Table 3). Previously, *in the presence of albumin* 300–600 μM LA concentrations were shown to depolarize mitochondria (de Oliveira et al., 2020b; Patel et al., 2016). In contrast, in the absence of albumin we note 2.5–30 μM *unbound* LA is sufficient to depolarize endothelial (HUV-EC) cell mitochondria (Figures 4A and 4B), which is reversed by albumin. This supports the deleterious role of unbound LA in causing endothelial injury (i.e., elevated E-selectin, ICAM-1; Figures 4C and 4E) in severe COVID-19 patients and in our mice. The endothelial injury was corroborated by electron microscopy (Figure 6O), elevated circulating PECAM-1 levels and higher PECAM-1 expression on IHC (Figures 6H and 6J). Such cell injury, along with previous studies showing LA to cause cytochrome c leakage, reduce ATP levels, inhibit mitochondrial complexes I and V (Khatua et al., 2019; Navina et al., 2011; Patel et al., 2016) may also explain the pulmonary and renal TUNEL positivity in LA treated mice (Figures 5E, 5F, 5H, and 5J), and perhaps in reports of COVID-19 patients (Li et al., 2020b; Liu et al., 2020; Santoriello et al., 2020). We also note that the renal tubule dilation (Figures 5E and 5H yellow arrows) is similar to that noted on COVID-19 autopsies (Santoriello et al., 2020). The renal and lung injuries are further corroborated by elevated BUN and creatinine (Figures 5C and 5D) along with elevated LDH and type-II pneumocyte injury markers (Figure 5K–5O) in the BALs of LA treated mice. Recently LA was shown to mediate the loss of transendothelial resistance and leakage of high molecular weight dextran across endothelial monolayers (El-Kurdi et al., 2020; Khatua et al., 2021). On electron microscopy we note this endothelial injury can disrupt vascular integrity (Figures 6O and 6P). These can explain the shock (Figure 5A) and hypoalbuminemia in severe COVID-19 and LA treated mice (Figures 2 and 3). Overall, the shock, renal and lung injury that we note in LA treated mice may explain the MOF noted in COVID-19 patients who also had elevated LA. The pro-inflammatory state triggered by LA is supported by the higher cytokines (Table 6) and hypothermia (Figure 5B) which is a part of the severe systemic inflammatory response syndrome (Leisman et al., 2020). Higher circulating DAMPs (e.g., ds-DNA, HMGB1; Figures 4G–4I), and endothelial proteins such as E-selectin (Figures 4C and 4D), PECAM-1 (Figure 6H), or ICAM-1 (Figures 4E and 4F) provide a mechanistic link for the prothrombotic state and hepatic portal thrombosis, pulmonary thrombosis induced by LA (Figures 6D and 6J), which is similar to COVID-19 autopsies (Diaz et al., 2020; Sonzogni et al., 2020). The LA induced increase in coagulation factor III (CFIII; Figure 6A), fibrinogen, plasminogen activation inhibitor-1 (PAI-1), TFPI and soluble PECAM-1/CD31 levels (Figures 6E–6H) are also noted in severe COVID-19 associated thrombosis (Friedrich et al., 2020; Hottz et al., 2020; Li et al., 2021; Lopez-Castaneda et al., 2021; White et al., 2021) as are the thrombocytopenia and an elevated activated clotting time (Figures 6K–6L) (Ackermann et al., 2020; Liu et al., 2020; Terpos et al., 2020). Lastly, the DAMPs we note increased, were previously shown to trigger the cytokine storm (e.g., HMGB1; Figure 4I) (Andersson et al., 2020; Hegyi et al., 2020; Yang et al., 2020), cause thrombosis (e.g., extracellular dsDNA, histone-DNA complexes; Figures 4G, 4H and 4J) (Fuchs et al., 2010; Semeraro et al., 2011) and result in a clinical picture of severe sepsis(Xu et al., 2009). Therefore, as summarized in Figure 8, these unbound LA induced phenomena can explain the inflammation, MOF and thrombosis (Figure 6) noted in severe COVID-19.

LA itself is a precursor of arachidonic acid (Gao et al., 2010), which is elevated in our COVID-19 ICU patients (Table 3), those with elevated IL-6 (Thomas et al., 2020), and mice given LA. Arachidonic acid can induce platelet aggregation, and increase the formation of prothrombotic thromboxane A2 (Lagarde et al., 2010), resulting in increased thrombosis.

There is a potential role for early albumin and calcium supplementation to prevent lipotoxicity in COVID-19. We have described experimentally what occurred when albumin was administered with LA and calcium: it inhibited the increase in unbound FA and prevented DAMP increase, coagulation abnormalities, and MOF development; resulting in improved survival in mice. The potential utility of albumin therapy in COVID-19 patients was recently described by an Italian group (Violi et al., 2021). In an observational prospective study performed in 29 SARS-CoV-2 patients treated with anticoagulant alone or anticoagulant plus albumin supplementation for 7 days, the investigators demonstrated a significant decrease of D-dimer only in the albumin-treated patients, who also had significantly reduced mortality (0/10 versus 8/19 without albumin supplementation; p = 0.02).

In summary, we note that during severe COVID-19 infection, the lipolytic release of UFAs like LA, perpetuated by the hypoalbuminemia and hypocalcemia induced by LA may result in cellular uptake of the unbound FAs like LA, resulting in mitochondrial injury. This injury to endothelial and other cells *in vivo* may result in shock, renal failure, DAMP release, and the consequent cytokine storm, MOF, and thrombosis that we note during severe COVID-19 infection.

### Limitations of the study

We acknowledge several limitations. First, our prospective cohorts are small, and the cohort-2 non-COVID-19 ICU patients are not an ideal control group because these included septic patients with whom they could share some pathophysiologic mechanisms. However, on excluding septic patients, the most important lipotoxic, inflammatory, and thrombotic abnormalities persisted. In addition, although organ failure was increased in the COVID-19 ICU group, mortality did not achieve statistical significance compared to the non-COVID group. To address this, we compare the NEFA profile between ICU survivors and non-survivors and note that NEFA, UFAs and specifically LA and unbound fatty acids were higher in non-survivors (Table S6). We also do not have an obese animal model of COVID-19 to test the hypothesis that unbound fatty acids worsen COVID-19 in these models, nor do we provide proof of albumin having a therapeutic role in these. This issue is partly addressed by the human study showing that COVID-19 patients receiving albumin and anticoagulation had improved survival than those who received anticoagulation only (Violi et al., 2021). Another limitation included the lack of lipase levels in the large retrospective cohort; however, large meta-analyses have shown lipase elevation to be associated with worse COVID-19 outcomes in the absence of clinical pancreatitis (Yang et al., 2022). Therefore, although this study provides preliminary evidence of and mechanisms supporting lipotoxic exacerbation of COVID-19, its findings need to be confirmed in larger studies.

## STAR★Methods

### Key resources table

**Table:** 

REAGENT or RESOURCE	SOURCE	IDENTIFIER
**Antibodies**		

Thyroid transcription factor 1	SantaCruz	AF594, 8G7G3/1, Cat# sc-53136, RRID:AB_793529
CD208	Biorbyt	CF647, orb665591
Anti-CD31/PECAM-1	SantaCruz	Clone H-3, Cat# sc-376764, RRID: AB_2801330

**Biological samples**		

COVID-19, ICU patient samples	Mayo Clinic Arizona	IRB approved

**Chemicals, peptides, and recombinant proteins**		

Annexin V apoptosis marker	ThermoFisher Scientific	A13201
FACS staining buffer	ThermoFisher Scientific	00422226
Coumarin-LA	Nanosyn Inc	Custom synthesized

**Critical commercial assays**		

Cytotoxicity Detection kit	Roche	04744942001
Total serum NEFA	Fujifilm Wako chemicals	LabAssay ™ NEFA kit
Unbound fatty acids	FFA Sciences	ADIFAB2 method
Human Cytokines-luminex assay	R&D Systems	Premixed Multi-Analyte Kit
ApopTag Peroxidase *In Situ*Apoptosis Detection Kit”	EMD Millipore	S7100
Mouse Cytokines-luminex assay MILLIPLEX MAP Mouse CVD Magnetic Bead Panel 1	Millipore	MILLIPLEX MAP

**Experimental models: Cell lines**		

HUVEC	Lonza	C2519A

**Experimental models: Organisms/strains**		

CD-1 mice	Charles River Labs	ICR

### Resource availability

#### Lead contact

Further information and requests for resources and reagents should be directed to and will be fulfilled by the lead contact, Dr. Vijay Prem Singh (singh.vijay@mayo.edu).

#### Materials availability

The availability of LA-coumarin is subject to Material transfer agreement.

#### Data and code availability

All de-identified data reported in this paper will be shared by the lead contactupon request. This paper does not report original code. Any additional information required to reanalyze the data reported in this paper is available from the lead contactupon request.

### Experimental model and subject details

#### Human studies

The age, sex of the subjects in the various cohorts in the study are mentioned in the respective Tables. The cohorts are detailed below.

##### Hospitalized pre-ICU COVID-19 cohort (cohort 1)

The study was from 5/12/2020, till 12/03/2020 on consented inpatients at Mayo Clinic Hospital (MCH; Phoenix, AZ), approved by the Mayo Foundation Institutional Review Board (IRB) and conformed to the regulatory standards of the institution. Inclusion criteria: ≥18 years old with positive SARS-CoV-2 PCR within 14 days, and sera available within 1 day of admission and dismissal to home (mild COVID) or ICU transfer (severe COVID).

##### Prospective ICU cohort (cohort 2)

Study was from May 1^st^, 2019 to October 31^st^, 2020 in a 30-bed multidisciplinary ICU at MCH, with IRB approval as above. Inclusion criteria: Consecutive critically ill patients ≥18 years of age admitted to the ICU; for COVID-19 patients, a positive SARS-CoV-2 PCR test within 14 days was required for inclusion. Exclusion criteria included DNR/DNI and comfort care patients, ICU readmissions, and patients who had not agreed to the use of their medical records for research. Data collection included patient characteristics, hospital course, laboratory values, and interventions on all enrolled patients during their hospitalization. The APS, APACHE IV score, and predicted hospital mortality rates based on these scores were calculated using an online APACHE IV calculator (Zimmerman et al., 2006). SOFA score (Moreno et al., 1999; Vincent et al., 1996) was documented daily from day 1 to day 7. Organ dysfunction was defined as a SOFA score of 1 or 2 points and organ failure as a SOFA score ≥3. MOF was defined as two or more organ failures (Moreno et al., 1999; Vincent et al., 1996). Interventions evaluated included use of renal replacement therapy (RRT), vasopressors, and extracorporeal membrane oxygenation (ECMO). Main outcomes evaluated included development of MOF, ICU length of stay (LOS), hospital LOS, mechanical ventilation days, hospital mortality, 28-day mortality, and development of thrombotic complications including deep vein thrombosis (DVT) and/or pulmonary embolism (PE).

##### Retrospective cohort (Cohort-3)

A retrospective review of all adult patients older than 18 years hospitalized with COVID-19 infection at Beaumont Health, an eight-hospital system in Southeast Michigan, USA between March 13 and May 5, 2020 was performed. Variables were abstracted utilizing automated reported generated by Toad Data Point multi-platform database query tool from Beaumont’s electronic medical record (EPIC System, Verona, WI, USA). A separate review of individual records was performed from all patients over 18 years of age hospitalized with a diagnosis of COVID-19 at University Hospital, an academic tertiary referral center associated with the University of Texas Health at San Antonio, Texas, USA between June 10 and September 11, 2020. This resulted in 3969 patients from 2 different health care systems. Variables were collected manually for all patients and included age, sex, race, body mass index (BMI), and comorbidities. Baseline laboratory values included serum creatinine, c-reactive protein, blood urea nitrogen (BUN), white blood cell count (WBC), platelet count (PLT). Additionally, serum calcium and albumin levels for the first 4 days of hospitalization were collected. The main outcome evaluated was in-hospital mortality. All data collection and chart review activities were appropriately vetted and approved by the IRBs of the respective institutions.

#### Animal studies

These were done in 8-12-week-old (30–40gm) Male, CD-1 mice from Charles River Laboratories (Wilmington, MA). Mice were fed a standard chow *ad libitum*, with full access to water, and were housed in standard cages with a normal day night cycle as before(El-Kurdi et al., 2020; Khatua et al., 2020, 2021). There were 4 groups: Controls, LA, PA treated and those administered LA with calcium and albumin (LA + Ca, Alb.). There were at least 7 mice per group. LA was given intraperitoneally at 0.2% body weight (El-Kurdi et al., 2020; Khatua et al., 2020, 2021). These mice received 1.0 mL saline subcutaneously 3 times per day. Co-administration of a similar does of LA with calcium and albumin (LA + Ca, Alb.) was done after gently mixing 500μL LA (at 37°C for 2 hours) with 10 mL of 25% albumin (fatty acid free in saline) containing 20mM calcium. This was delivered intraperitoneally at 1.2mL/30 gm body weight. PA (333 mg/kg) was administered intraperitoneally either alone via a sterile peritoneal incision, or via intraperitoneal injection having PA dissolved in 50% dimethyl sulfoxide. While some mice overlapped with previous cohorts (El-Kurdi et al., 2020; Khatua et al., 2020, 2021), the current studies were separate from previous ones reported. Mice were monitored daily thereafter for general appearance, grooming, posture, activity, food intake, and vitals for the next 3 days. Rectal temperature was measured with a clinical thermometer; carotid artery pulse distention was measured using a neck collar of a MouseOx pulse oximeter (Starr Life sciences, Oakmont, PA). Mice were euthanized on day 3 or when unable to ambulate, moribund or if noted to be in distress (El-Kurdi et al., 2020). Vitals prior to this euthanasia are the ones reported. Blood parameters measured were the ones at the time of euthanasia. Creatinine, ionized calcium, and BUN were measured using the CHEM8+ cartridge in the i-STAT 1 blood analyzer (Abbott Point of Care, Orlando, Florida, USA). All protocols were approved by the institutional animal care and use committee of the Mayo Clinic Foundation.

#### Bronchoalveolar lavage (BAL) analysis

BAL was performed via tracheal incision at the time of euthanasia. To collect mice lungs BAL fluid, an incision was made on the disinfected neck skin. The trachea was exposed by blunt dissection. After tracheal incision with a scalpel, a catheter was placed in the exposed trachea. which was connected to a syringe filled with 1 mL of sterile saline solution. A cotton ligature was placed around the trachea and catheter to avoid flowing back of the fluid to the upper airways. The lungs were flushed with saline gently, preventing the collapse of the lung airway. The aspirated fluid was collected and centrifuged (400g, 10 min, 4°C) to pellet the cells, which were immediately as below. The supernatant was analyzed for lactate dehydrogenase (LDH) leakage and total proteins. A colorimetric LDH leakage assay was performed with Roche Cytotoxicity Detection kit (04744942001) following manufacturer’s protocol. The data obtained was used to calculate LDH activity (U/L). Total protein was estimated by using Pierce BCA Protein Estimation kit (23225) following manufacturer’s protocol and reported as mg/mL. For flow cytometry the pelleted cells were fixed (20 min, RT) in BD FACS lysing solution (349202), followed by staining with antibodies Thyroid transcription factor 1 (AF594; SantaCruz 8G7G3/1), CD208 (CF647; Biorbyt orb665591), and Annexin V apoptosis marker (AF488; Invitrogen 13201) in FACS staining buffer (Invitrogen™ 00422226) for 40 min in dark on ice. Protocols followed were as recommended by the respective manufacturers. The stained cells were washed (400g, 5min) 3 times and transferred in FACS tubes for analysis. 25,000 counts were read per sample with BD FACS Fortessa Instrument (BD biosciences) and the data acquired was analyzed using Flow Jo software (BD).

#### HUV-EC cell culture

HUV-EC-C cells from AmericanType Culture Collection (ATCC; Manassas, VA) were cultured in Kaighn's Modification of Ham's F-12 Medium (F-12K: ATCC, Manassas, VA) supplemented with 10% Fetal Bovine Serum (FBS: ATCC, Manassas, VA), 1% penicillin/streptomycin (Life Technologies, Carlsbad, CA), 0.1 mg/mL Heparin (Millipore Sigma, St. Louis, MO) and 50 μg/mL Corning Endothelial Cell Growth Supplement (ECGS: Fisher Scientific) (El-Kurdi et al., 2020). Before use the cells were transferred to HEPES buffer pH 7.4 (20 mmol/L HEPES, 120 mmol/L NaCl, 5 mmol/L KCl, 1 mmol/L MgCl2, 10 mmol/L glucose, 10 mmol/L sodium pyruvate) and used as described in the section for imaging studies below.

### Methods details

#### Reagents

Linoleic acid ≥99% purity was procured from Sigma-Aldrich and stored at −80C. A fresh vial was used for each study. All reagents were of the highest purity and procured from the specified manufacturer. Routine chemicals were obtained from Sigma-Aldrich (Saint-Louis, MO).

#### Blood sample handling of prospective cohorts-1 and -2

Serums samples were collected within 12 hours of ICU admission. Serum was collected using tubes with serum separators. The samples were stored, transported at 4°C and analyzed or frozen at −80°C within the next 12 hours. One sample of the 8 severe COVID-19 patients belonging to cohort 1 was spilled and lost and not available for serum FA analysis. Serum FAs were analyzed by Gas chromatography at the Vanderbilt University medical center lipidomics core (de Oliveira et al., 2020b).

#### Unbound fatty acids using ADIFAB reagent

Total serum unbound FAs were measured using the fluorescent ADIFAB2 method (FFA sciences; San Diego, CA) using the manufacturer’s instructions as reported in published studies (Khatua et al., 2021). A calibration curve using oleic acid standards (100nM to 50 μM) in DMSO was used as a reference. Figure S1 shows a summary of 9 calibration curves as black dots, with the mean depicted as the dashed line. The red dots show the values of unbound oleic acid standard supplied by the manufacturer. Based on these calibration curves and standards, low and high serum controls were generated and used for quality control for each experiment. These fluorescent unbound fatty acid results are shown for all studies. In case of quantification of specific unbound fatty acids (for cohort 1) using gas-chromatography mass-spectrometry, the following protocol was used.

#### Quantification of unbound fatty acids using gas-chromatography mass spectrometry (GC-MS) for cohort-1

*Dealbumination*: Patient sera that were to be de-albuminated were thawed and vortexed. The sample to be de-albuminated was removed and warmed to 37°C. This temperature is important to keep unbound fatty acid behavior relevant to normal human body temperature. Dealbumination was done using the Pierce^TM^ Albumin depletion kit (Thermo Scientific, Rockford, IL) using the manufacturer’s instructions with all steps at 37°C and the following modifications. Each sample was re-applied to and passed through a column 3 times. This was repeated through two consecutive columns to ensure complete de-albumination. Albumin was measured on the final flow through collected using the bromo cresol green reagent (BCG) to ensure complete dealbumination (please see Figure S2 after the supplementary Tables). Lipids were then extracted from the final flow through using the Folch method(Folch et al., 1957). At the end of extraction, the chloroform was evaporated under nitrogen and the samples were then resuspended by sonication in 0.2 mL PBS, pH 7.4. Part of this was processed for Gas-chromatography mass spectrometry as per the protocol of Kangani et al.(Kangani et al., 2008), and described in the section below:

1. Fatty acid extraction: Kangani and Delany PlasmaFree Fatty Acid Extraction
  i. Add 5μL of internal standard (in standards section) to the 160μL of de-albuminated dried Folch extract resuspended in PBS (equivalent to 44μL of serum).
  ii. Add 2mL of a 40:10:1, Isopropanol: Hexane: Hydrochloric acid (1M) mixture for each sample and transfer to a 13mL glass tube with PTFE lined screw caps using a pasteur pipette. Mix 50mg of BHT per liter of the 40:10:1 solvent mixture.
  iii. Thoroughly vortex mixture for 30 minutes followed by a 10-minute incubation at room temperature.
  iv. Add 1.89mL of LCMS grade water then 4mL of hexane to each tube and vortex for 5 minutes.
  v. Centrifuge at 1000g for 10 minutes at 4°C
  vi. Transfer the hexane upper phase to a new glass tube using a pasteur pipette, then evaporate using nitrogen and no heat.
2. Deoxo-Fluor Derivatization
  vii. On ice, add 200μL of dichloromethane to all the vials, followed by 4μL of diisopropylethylamine, then 8μL of dimethylamine.
  viii. Add 2μL of Deoxo-Fluor to the wall of the vial, immediately cap and vortex for 5 seconds to mix.
  ix. Incubate the samples at −20°C for 5 minutes, then at room temperature for 10 minutes.
  x. Transfer the samples to screw top glass tubes with PTFE lined screw caps containing 2mL of LCMS grade water, vortexing briefly to stop the reaction.
  xi. Add 4mL of hexane and vortex the mixture for 15 minutes.
  xii. Centrifuge the mixture at 3000 RPM for 10 minutes.
  xiii. Transfer the organic upper phase to a new test tube using a pasteur pipette and evaporate using nitrogen and heat at 40°C.
  xiv. Resuspend in 200μL of hexane with caffeine and transfer to an auto-sampler vial.
3. GC-MS

The GC is an Agilent GC 7890B system with an Agilent 5977A MSD (single quadrupole) attached. The MS source is set at 275°C whereas the quad is set at 150°C. The carrier gas of Helium has a flow set at 1 mL/min. The injection volume is set at 1μL with the injector port temperature set at 260°C. The oven is programmed to begin at 140°C for 2 minutes, with a ramp up at 20°C/min to 200°C which is held for 4 minutes. A second ramp is started at 5°C/min up to 260°C with no holding time, where the final ramp beings at 10°C/min up to a maximum of 300°C that is held for 15 minutes. The GC is equipped with a capillary column from Agilent (HP-5MS UI, 30 m × .25 mm I.D with a .25um film thickness).

4. Use of standards
  xv. A standard curve is taken through the entire extraction and derivatization process that is made up of a combination of C12:0, C14:0, C16:0, C16:1, C18:0, C18:1, C18:2, C18:3 and C20:4 in heptane. The curve was made to 20μM and was diluted in a serial dilution to 10μM, 5μM, 3μM, 2μM, 1μm and 0.5μM the curve also includes the internal standards.
  xvi. A mixture of Internal standards of C16:2, C17:0, and C19:2 is added to every sample at 4.545μM, 9.09μM and 9.09μM respectively and used to determine extraction efficacy.
  xvii. Caffeine is used at 5ppm as a GC standard
5. Calculation of results
  xviii. Extraction blanks of hexane are subtracted out of the GC response and then each individual fatty acid is calculated for every sample from solving a linear equation generated from the standard curve. The values are then adjusted accounting for the extraction efficacy of each internal standard value targeting the theoretical values of 4.545 and 9.09μM.

#### Assays

*DAMPs*: ds-DNA was measured using the Quant-iT PicoGreen dsDNA reagent (Life Technologies, Carlsbad, CA). Histone complex DNA fragments were measured using a kit from Sigma-Aldrich (Saint-Louis, MO), or HMGB-1 from FineTest (Wuhan Fine Biotech Co., Ltd, Wuhan, China) were measured using ELISA.

*Cytokines and coagulation relevant molecules*: In humans CXCL1 (C-X-C Motif Chemokine Ligand 1), Interleukin-1 beta [IL-1β], IL-1Rα, IL-4, IL-6, IL-8, IL-10, IL-18, CXCL10, monocyte chemotactic protein [MCP-1] and tumor necrosis factor- α (TNF-α) were measured and analyzed using MILLIPLEX Human cytokine/ Chemokine/ Growth Factor Panel A (Millipore, Burlington, MA, USA), and sE-selectin, Soluble intercellular adhesion molecule-1 (sICAM-1), coagulation factor III (or tissue factor), and tissue factor pathway inhibitor (or TFPI) were measured and analyzed using Luminex Assay, Human Premixed Multi-Analyte Kit (R&D Systems, Minneapolis, MN, USA).

In mice CXCL1, IL-1β,IL-4, IL-6, IL-10, IL-18, IP-10, MCP-1, TNF-α, sE-selectin, s-ICAM1, PECAM-1 and PAI-1 were measured using MILLIPLEX MAP Mouse Cytokine/Chemokine Magnetic Bead Panel (Millipore, Burlington, MA, USA) and MILLIPLEX MAP Mouse CVD Magnetic Bead Panel 1 (Millipore, Burlington, MA, USA) according to manufacturer’s recommendations on a Luminex 200 System (Life Technologies, Carlsbad, CA, USA) and analyzed using the xPONENT software (de Oliveira et al., 2020b; Noel et al., 2016).

*Biochemical and hematological assays*: The biochemical assays (lipase, BUN [blood urea nitrogen], Calcium, albumin; Pointe Scientific, Canton, MI, USA) were done as per the manufacturer’s protocol(de Oliveira et al., 2019, 2020a, 2020b; Noel et al., 2016; Patel et al., 2015). Total serum NEFA were measured LabAssay ™ NEFA kit from Fujifilm Wako chemicals (https://labchem-wako.fujifilm.com/us/product/detail/W01S10LABNEFA-M1.html) which requires only 4 μL of sample. Individual C12-C18 fatty acids were measured using Gas chromatography-Mass spectrometry (Kangani et al., 2008) using 25 μL of sample.

*Activated clotting time (ACT)*: ACT were measured from freshly drown cardiac blood by using i-STAT Celite ACT Cartridge on i-STAT1 analyzer (Abbott Point of Care Inc, Princeton, NJ, USA).

*Platelet counts*: Freshly isolated mouse blood was diluted with a 1% ammonium oxalate solution in 1:20 ratio and allowed 15–20 minutes to lyse all erythrocytes while the leukocytes, platelets, and reticulocytes remain intact. The solution was placed on Hemocytometer and put cover glass on it. The entire large center square was counted and multiplied by 200 to calculate the platelet count per microliter.

*TFPI and fibrinogen*: Mouse TFPI (Antibodies-online Inc, Limerick, PA, USA) and Fibrinogen (ISL Inc, Portland, OR, USA) ELISA were done from mouse serum as per manufacturer protocol.

*Histologic analysis*: Lung and liver tissue of mice were procured at the time of necropsy (CO2 euthanasia) and were fixed with 10% neutral buffered formalin (Fisher Scientific) and embedded in paraffin and sectioned. Liver paraffin section (5 microns) slides stained by hematoxylin and eosin (H&E) were used to identify portal venous thrombi by a pathologist (S. N.) blinded to the treatment received. TUNEL staining of the lungs and kidneys were done using the ApopTag Peroxidase *In Situ*Apoptosis Detection Kit” from EMD Millipore (Catalog number #S7100) as per validated manufacturer’s protocol(de Oliveira et al., 2020b; Durgampudi et al., 2014; Noel et al., 2016), and Imaged at 40x with 7–15 fields photographed per mouse, and TUNEL positive brown staining was quantified and averaged for each mouse in a blinded fashion (B.K).

*PECAM-1 IHC: PECAM-1*: Lungs paraffin section (5 microns) slides were stained with Anti-CD31/PECAM-1 antibodies (Clone H-3, Catalog numbersc376764, Santa Cruz Biotechnology, Dallas, Texas, USA; 1:200) at the Pathology Research Core at Mayo Clinic Rochester, MN.

#### *In vitro* studies

All data shown are representative of 5 independent experiments. For mitochondrial depolarization studies and mitochondrial uptake studies we show representative curves and images respectively.

*Lipid studies:* Linoleic acid was probe sonicated into media in two-steps, and kept warm at 37°C without any organic solvent based on publications that have utilizing this (Khatua et al., 2019, 2021) to ensure a stable emulsion for the duration of *in vitro* studies. LA-coumarin was custom synthesized by reacting Linoleic acid alkyne with 7-hydroxycoumarin azide at Nanosyn Inc (Santa Clara, CA). Structure was confirmed by LC/MS and purity determined by HPLC/UV was >95% with <1% hydroxycoumarin.

*Isothermal titration calorimetry*: The interaction of calcium or albumin with LA was carried out as described elsewhere(El-Kurdi et al., 2020; Khatua et al., 2020). Briefly, the interactions were studied on a Nano ITC (TA Instruments-Waters LLC, New Castle, DE, USA) instrument using Nano ITCRunSoftware v3.5.6. A total of 25 aliquots of freshly prepared, degassed calcium chloride (1 mM in 10 mM HEPES [4-(2-hydroxyethyl)-1-piperazineethanesulfonic acid], pH 7.4; 1.7 μL per injection) were injected into degassed LA (0.5 mM in 10 mM HEPES, pH 7.4). Similarly, albumin (stock 0.85mM) was injected into LA (1 mM), or LA (1mM stock) into albumin (0.16 mM) at 37°C. The thermodynamic parameters (Kd, n and ΔH) for calcium–LA and albumin–LA was calculated from NanoAnalyze Software v3.10.0 (TA Instruments-Waters LLC, New Castle, DE, USA). Parameters were calculated from 4-6 different experiments and represented as Mean ± SEM.

*Live imaging studies:* HUV-EC-C cells were grown as monolayers on 35 mm cell culture dish with 20 mm glass bottom (Cellvis, Mountain View, CA) after coating with type I collagen (BD Bioscience, Two Oak Park, Bedford, MA). Cells were loaded with MitoTracker- Red CMXRos (Excitation 580nm, Emission 600nm; ThermoFisher) in a complete F-12K Medium at 37 °C for 30 min and then washed twice with HEPES buffer pH 7.4 (20 mM HEPES, 120 mM NaCl, 5 mM KCl, 10 mM glucose, 10 mM sodium pyruvate) immediately before use. Imaging was done in HEPES on a 37°C warmed stage of a laser scanning confocal microscope (LSM 800 ZEISS) using a 63x Plan-Fluor oil immersion objective. For localization of LA, cells were exposed to LA (30μM) along with tracer amounts (1.5 μM) of LA-coumarin after 1 minute. Images were collected at 30 second intervals. LA coumarin was visualized with excitation of 400nm and emission 450nm. 0.1% Alexa 647 conjugated Fatty acid free albumin (ThermoFisher) was added after 180 seconds and imaging (excitation at 640nm and emission 660nm) was continued for 5 minutes.

*Mitochondrial membrane potential*: HUV-EC-C cells were harvested by trypsinization, washed with warm complete F-12K Medium, loaded with 5,5’,6,6’-Tetrachloro-1,1’,3,3’-tetraethylbenzimidazolylcarbocyanine iodide (JC-1, 5 μg/mL; Enzo Life Sciences, Farmingdale, NY) in the media and incubated at 37°C for 30 minutes in the dark. After cells were washed and re-suspended in HEPES buffer pH 7.4(Khatua et al., 2020; Navina et al., 2011; Patel et al., 2016; Singh et al., 2009; Singh and McNiven, 2008) and stored on ice till the assay was conducted. LA was added after 100 seconds at the indicated concentrations to the stirred cell suspensions in a quartz cuvette at 37°C. Mitochondrial inner membrane potential (Ψm) was determined by excitation at 490nm, and alternate measuring emission at 510 and 590 nm using the F2100 Hitachi Fluorescence Spectrophotometer. The data were collected every 10 seconds. The net increase over time point before addition was plotted vs. time. The increase in 510/590 emission ratio was used as a measure of mitochondrial depolarization.

### Quantification and statistical analysis

Continuous parametric variables were reported as means and standard deviations while non-parametric continuous variables were reported as medians and interquartile ranges. Unpaired Student’s t tests were used to compare continuous variables with normal distribution and the Wilcoxon rank test for skewed distribution. ANOVA with multiple comparisons was used for comparisons of multiple groups. For comparison of categorical variables, chi-square tests were used if the number of elements in each cell was ⩾ 5; Fisher’s exact test were used otherwise. A p value ⩽ 0.05 was considered statistically significant. Univariate and multivariate regression analyses for the retrospective cohort study were conducted for the baseline variables abstracted identifying associations between variables and in-hospital mortality. All data analyses were performed using IBM SPSS Statistics for Windows (Version 23.0. Armonk, NY: IBM Corp, released 2015), and JMP Statistical Software (Version 14.1.0; SAS Institute, Cary, NC, USA).
