# Supplementary material for: Evidence showing lipotoxicity worsens outcomes in covid-19 patients and insights about the underlying mechanisms
Source: iScience. 2022 Apr 27;25(5):104322. doi: 10.1016/j.isci.2022.104322 (PMC9045865; doi:10.1016/j.isci.2022.104322)
Supplement: Document S1. Figures S1 and S2 and Tables S1–S24 [file mmc1.pdf]

iScience, Volume *25*

Supplemental information

Evidence showing lipotoxicity worsens outcomes in covid-19 patients and insights about the underlying mechanisms

Rodrigo Cartin-Ceba, Biswajit Khatua, Bara El-Kurdi, Shubham Trivedi, Sergiy Kostenko, Zaid Imam, Ryan Smith, Christine Snozek, Sarah Navina, Vijeta Sharma, Bryce McFayden, Filip Ionescu, Eugene Stolow, Sylvia Keiser, Aziz Tejani, Allison Harrington, Phillip Acosta, Saatchi Kuwelker, Juan Echavarria, Girish B. Nair, Adam Bataineh, and Vijay P. Singh

SUPPLEMENTARY MATERIAL

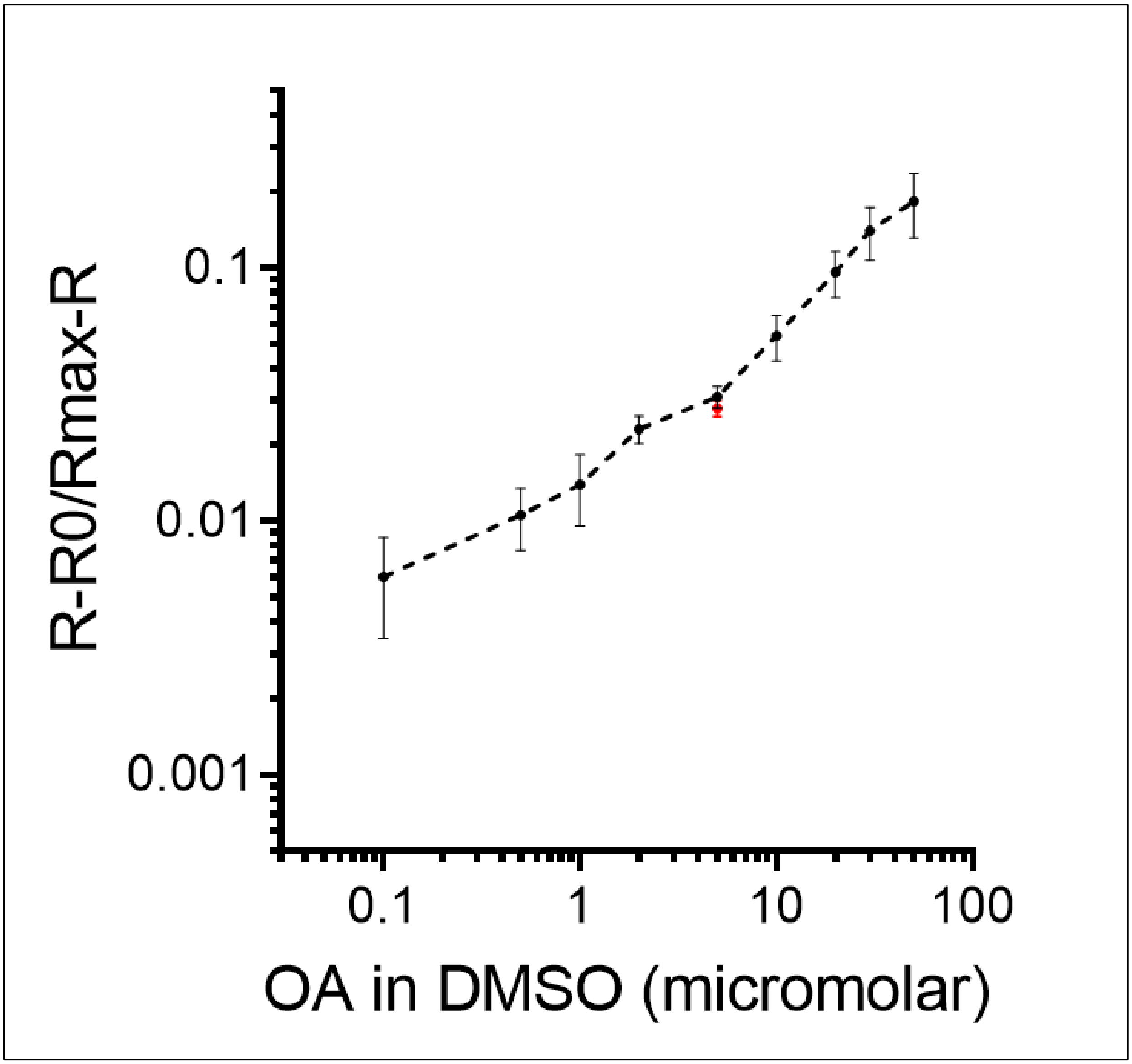

Figure S1: Calibration curve for ADIFAB reagent using oleic acid standards in dimethyl sulfoxide (DMSO), related to measurement of unbound fatty acids using ADIFAB reagent in STAR methods: Calibration curves for oleic acid standards in DMSO showing the results of 9 different experiments in black dots and the dashed line connects the means for each concentration. The red dot shows the value of the unbound oleic acid standard supplied by the manufacturer. Error bars depict standard error of mean.

Albumin values (BCA assay) before and after albumin depletion using 2 columns of the Pierce™ Albumin depletion kit

Absorbance

	1	2	3	4	5	6	7	8	9
A	0.286	0.289	0.274	0.263	0.150	0.152	0.154	0.154	0.263
B	0.274	0.237	0.291	0.276	0.147	0.145	0.146	0.149	0.264
C	0.273	0.296	0.242	0.259	0.141	0.147	0.147	0.148	0.269
D	0.314	0.303	0.233	0.268	0.145	0.149	0.142	0.148	
E	0.284	0.262	0.267	0.257	0.151	0.149	0.147	0.148	
F	0.269	0.293	0.280	0.245	0.150	0.147	0.159	0.147	
G	0.295	0.290	0.281	0.160	0.148	0.150	0.148	0.177	
H	0.260	0.270	0.273	0.154	0.146	0.147	0.152	0.162	

Sample #s

	Before albumin depletion				After albumin depletion				
A	4	24	48	22	4	24	48	22	4g/dl BSA
B	6	26	50	28	6	26	50	28	4g/dl BSA
C	8	32	52	30	8	32	52	30	4g/dl BSA
D	12	38	54	34	12	38	54	34	
E	14	40	56	36	14	40	56	36	
F	16	42	58	60	16	42	58	60	
G	18	44	2	Blank	18	44	2	Blank	
H	20	46	10	Blank	20	46	10	Blank	

Absorbance minus blank

Minus Blank								
0.127	0.130	0.115	0.104	-0.009	-0.007	-0.005	-0.005	0.104
0.115	0.078	0.132	0.117	-0.012	-0.014	-0.013	-0.010	0.105
0.114	0.137	0.083	0.100	-0.018	-0.012	-0.012	-0.011	0.110
0.155	0.144	0.074	0.109	-0.014	-0.010	-0.017	-0.011	
0.125	0.103	0.108	0.098	-0.008	-0.010	-0.012	-0.011	
0.110	0.134	0.121	0.086	-0.009	-0.012	0.000	-0.012	
0.136	0.131	0.122	0.001	-0.011	-0.009	-0.011	0.018	
0.101	0.111	0.114	-0.005	-0.013	-0.012	-0.007	0.003	

Albumin Conc.

Albumin(g/dl)							
4.78	4.91	4.35	3.91	-0.35	-0.25	-0.20	-0.19
4.33	2.95	4.98	4.41	-0.43	-0.52	-0.48	-0.37
4.32	5.16	3.14	3.77	-0.67	-0.45	-0.47	-0.40
5.84	5.44	2.80	4.10	-0.52	-0.38	-0.66	-0.41
4.70	3.89	4.09	3.70	-0.30	-0.38	-0.45	-0.41
4.15	5.05	4.58	3.26	-0.33	-0.44	0.00	-0.45
5.13	4.96	4.61		-0.40	-0.34	-0.43	
3.81	4.17	4.31		-0.49	-0.44	-0.27	

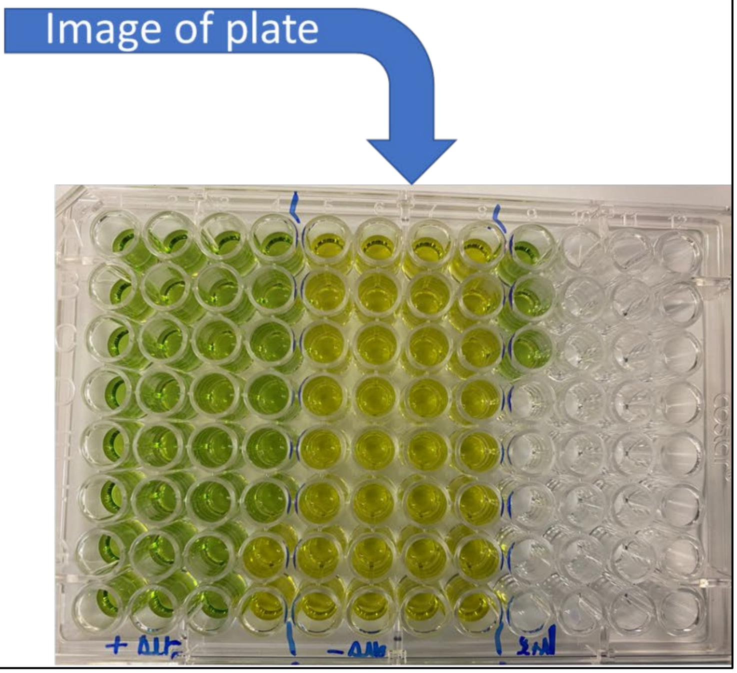

Figure S2: Results of serum dealbumination: Related to quantification of unbound fatty acids using gas-chromatography mass spectrometry in STAR methods Image of the excel (left side) showing measured absorbances and calculated albumin concentrations (Conc.) along with the plate used for the assay (right side). One the left side of each excel table is the title of what the table depicts. Columns

TABLE S1: Clinical characteristics comparing COVID versus non-COVID, non-septic shock patients admitted to the ICU related to table 2.

Variable	Non-COVID, Non-septic shock (49)	COVID-19 (39)	P value
Age in years, median (IQR)	67 (57-75)	55 (44-64)	< 0.0001
Female sex, n (%)	12 (24.5)	17 (43.6)	0.07
BMI in Kg/m^2, median (IQR)	27.8 (25.1-32.5)	30.2 (26.8-35.8)	0.04
Race, n (%)			< 0.0001
Caucasian	42 (85.7)	11 (28.2)	
Hispanic	4 (8.3)	8 (20.5)	
Asian	2 (4)	2 (5.1)	
African American	1 (2)	1 (2.6)	
Native American	0 (0)	17 (43.6)	
Diabetes Mellitus, n (%)	12 (24.5)	19 (48.7)	0.02
Hypertension, n (%)	25 (51)	16 (41)	0.39
Coronary artery disease, n (%)	23 (47)	5 (12.8)	0.001
Cancer, n (%)	5 (10)	2 (5)	0.45
Cerebrovascular disease, n (%)	4 (8)	3 (7.7)	0.99
Cirrhosis, n (%)	4 (8)	0 (0)	0.12
Chronic kidney disease, n (%)	7 (14)	3 (7.7)	0.50
Immunosuppression, n (%)	2 (4)	6 (15)	0.13
Pre-ICU days, median (IQR)	0 (0-2)	1 (1-3)	0.0003
ICU source, n (%)			< 0.0001
Emergency Room	8 (16)	3 (7.7)	
Hospital Ward	3 (6)	21 (54)	
Operating Room	36 (73.5)	0 (0)	
Outside hospital	2 (4)	15 (38.5)	
Temperature in °Celsius, median (IQR)	37.4 (35.9-38.3)	37.5 (37-38.2)	0.13
Median arterial pressure in mmHg, median (IQR)	61 (58-66)	69 (62-77)	0.005
Heart rate, median (IQR)	94 (84-103)	98 (86-108)	0.30
Respiratory rate, median (IQR)	22 (19-25)	29 (24-32)	< 0.0001
Fluids in first 24 hours, median (IQR)	5123 (2923-6183)	2001 (1319-2638)	< 0.0001
Urine output, first 24 h, median (IQR)	1821 (1392-2537)	1475 (920-2265)	0.15
Fluid balance 24 hours (mL), median (IQR)	3185 (621-4330)	169 (-765-1071)	< 0.0001
Glasgow Coma Scale, median (IQR)	12 (9-15)	15 (15-15)	0.001
Acute physiologic score, median (IQR)	39 (30-48)	38 (25-62)	0.97
APACHE IV, median (IQR)	52 (39-63)	51 (34-69)	0.57
Predicted mortality, median (IQR)	2.8 (1.1-9.4)	14.7 (7.3-37.6)	< 0.0001
Vasopressors on Day 1	27 (55)	17 (44)	0.39
SOFA score Day 1, median (IQR)	7 (5-9)	5 (4-8)	0.26
SOFA score Day 2, median (IQR)	5 (3-7)	5 (3-8)	0.72
SOFA score Day 3, median (IQR)	5 (3-7)	5 (3-8)	0.69
SOFA score Day 4, median (IQR)	4 (3-9)	6 (3-9)	0.39
SOFA score Day 5, median (IQR)	4 (2-11)	6 (4-10)	0.45
SOFA score Day 6, median (IQR)	7 (3-12)	6 (4-9)	0.84
SOFA score Day 7, median (IQR)	10 (3-13)	7 (4-10)	0.45
**Baseline laboratory results**			
White blood count (x10^9/L), median (IQR)	9.5 (7.5-12)	9.4 (5.2-15)	0.69

Platelets (x10^9/L), median (IQR)	127 (95-213)	226 (176-284)	< 0.0001
Sodium (mmol/L), median (IQR)	141 (138-142)	136 (133-138)	< 0.0001
Creatinine (mg/dL), median (IQR)	1.02 (0.76-1.2)	1.06 (0.81-1.68)	0.32
Blood urea nitrogen (mg/dL), median (IQR)	15 (12-21)	18 (12-35)	0.26
Glucose (mg/dL), median (IQR)	157 (132-177)	134 (107-210)	0.38
pH, median (IQR)	7.4 (7.35-7.43)	7.39 (7.31-7.44)	0.84
Lactate (mmol/L), median (IQR)	1.4 (1.1-2.3)	1.2 (1.1-1.7)	0.12
Bilirubin (mg/dL), median (IQR)	1 (0.5-1)	0.4 (0.3-0.6)	< 0.0001
Hematocrit (%), median (IQR)	29 (27-34)	38 (33-41)	< 0.0001
Lipase (U/L), median (IQR)	21.5 (11-45.7)	73.1 (42.2-121.9)	< 0.0001
Albumin (g/dL), median (IQR)	4 (3.8-4.3)	3.2 (2.9-3.4)	< 0.0001
Calcium (mg/dL), median (IQR)	8.7 (8.1-9.3)	8 (07.4-8.3)	< 0.0001
Ionized Calcium (mg/dL), median (IQR)	4.7 (4.3-4.9)	4.3 (4.1-4.5)	0.001

IQR: interquartile range, n: number, BMI: body mass index, ICU: intensive care unit, APACHE: Acute Physiology and Chronic Health Evaluation, SOFA: Sequential Organ Failure Assessment.

<u>TABLE S2:</u> Clinical characteristics and outcomes of 39 patients with COVID-19 admitted to the ICU comparing Caucasians versus non-Caucasian patients, related to table 2.

Variable	Caucasian (11)	Non-Caucasian (28)	P value
Age in years, median (IQR)	59 (52-70)	49 (44-63)	0.15
Female sex, n (%)	5 (45)	12 (43)	0.99
BMI in Kg/m^2, median (IQR)	31 (27-34)	30 (26-36)	0.82
Diabetes Mellitus, n (%)	6 (54)	13 (46)	0.73
Hypertension, n (%)	5 (45)	11 (39)	0.74
APACHE IV, median (IQR)	51 (34-66)	50 (33-74)	0.77
Predicted mortality, median (IQR)	14.7 (7-25)	14 (7-44)	0.85
Vasopressors on Day 1	4 (36)	13 (46)	0.72
28 day Mortality, n (%)	2 (18)	3 (11)	0.60
Venous thromboembolic events, n (%)	2 (18)	8 (28)	0.69
RRT, n (%)	2 (18)	7 (25)	0.99
MOF development, n (%)	5 (45)	18 (64)	0.3
**Baseline laboratory results**			
Lactate (mmol/L), median (IQR)	1.4 (1-1.9)	1.3 (1.1-1.8)	0.74
IL-6 (pg/ml), median (IQR)	355 (111-641)	363 (59-666)	0.70
TNFα (pg/ml), median (IQR)	32.7 (28.3-63.9)	41 (26.5-57.3)	0.98
Lipase (U/L), median (IQR)	109 (57-212)	65 (36-98)	0.07
Albumin (g/dL), median (IQR)	3.3 (3-3.5)	3.1 (2.5-3.4)	0.16
Calcium (mg/dL), median (IQR)	8.2 (7.4-8.4)	8 (7.4-8.2)	0.61
Ionized Calcium (mg/dL), median (IQR)	4.3 (4-4.4)	4.3 (4.1-4.6)	0.74
Unbound FA, median (μM)(IQR)	5.9 (4.3-10.9)	4.3 (2.3-7.8)	0.24
Linoleic acid (C18:2), median (μM)(IQR)	140 (81-191)	106 (72-146)	0.26

IQR: interquartile range, n: number, BMI: body mass index, ICU: intensive care unit, APACHE: Acute Physiology and Chronic Health Evaluation, RRT: renal replacement therapy, MOF: multiorgan failure

<u>TABLE S3:</u> Clinical characteristics and outcomes of 39 patients with COVID-19 admitted to the ICU comparing Native-Americans (NA) versus non-NA, related to table 2

Variable	Native-American (17)	Non-Native-American (22)	P value
Age in years, median (IQR)	61 (48-67)	51 (43-62)	0.19
Female sex, n (%)	7 (41)	10 (45)	0.99
BMI in Kg/m^2, median (IQR)	29.4 (26.6-34.8)	31.3 (26.7-37.9)	0.55
Diabetes Mellitus, n (%)	10 (59)	9 (41)	0.34
Hypertension, n (%)	8 (47)	8 (36)	0.45
APACHE IV, median (IQR)	41 (31-63)	54 (35-77)	0.32
Predicted mortality, median (IQR)	11 (6-33)	18 (9-40)	0.32
Vasopressors on Day 1	6 (35)	11 (50)	0.51
28 day Mortality, n (%)	3 (18)	2 (9)	0.63
Venous thromboembolic events, n (%)	5 (29)	5 (23)	0.72
RRT, n (%)	4 (24)	5 (23)	0.99
MOF development, n (%)	11 (65)	12 (55)	0.74
**Baseline laboratory results**			
Lactate (mmol/L), median (IQR)	1.2 (1.1-1.5)	1.7 (1-1.9)	0.41
IL-6 (pg/ml), median (IQR)	367 (206-627)	322 (51-717)	0.47
TNFα (pg/ml), median (IQR)	40.4 (27-55.7)	50.3 (28-66)	0.68
Lipase (U/L), median (IQR)	64 (34-93)	84 (48-185)	0.11
Albumin (g/dL), median (IQR)	3.1 (2.7-3.4)	3.2 (2.9-3.5)	0.52
Calcium (mg/dL), median (IQR)	8 (7.8-8.3)	8 (7.4-8.3)	0.56
Ionized Calcium (mg/dL), median (IQR)	4.5 (4.3-4.6)	4.1 (4-4.4)	**0.02**
Unbound FA, median (μM)(IQR)	4.2 (2.7-6.8)	5.7 (1.7-10.6)	0.42
Linoleic acid (C18:2), median (μM)(IQR)	108 (80-139)	117 (76-195)	0.42

IQR: interquartile range, n: number, BMI: body mass index, ICU: intensive care unit, APACHE: Acute Physiology and Chronic Health Evaluation, RRT: renal replacement therapy, MOF: multiorgan failure

<u>TABLE S4:</u> Fatty acid profile (in micromolars) of ICU patients comparing COVID versus non-COVID patients without septic shock as well as experimental animal data comparing control mice versus mice treated with linoleic acid (LA), related to table 3

Fatty acids (μM unless specified)	Non-COVID, Non-Septic shock (49)	COVID-19 (39)	P value	Control mice	LA mice	P value
Myristic acid	7.6 (5.7-10.7)	6.7 (5.3-10)	0.2	5.9 (4.9-7.8)	6.7 (6.1-8.3)	0.94
Palmitic acid	136 (116-178)	151 (116-217)	0.23	159 (148-200)	177(156-182)	0.23
Palmitoleic acid	14.8 (8.4-22.4)	16.8 (9.7-24.1)	0.38	16.9 (9.1-25.9)	20.4 (18.1-22.9)	0.62
Stearic acid	51.3 (38.4-58.7)	47.4 (38.4-56.1)	0.51	47.8 (38.3-57.9)	33.1 (29.5-42.0)	0.14
Oleic acid	226 (173-281)	243 (190-310)	0.25	75.1 (65.0-83.5)	99.0 (75.5-123)	0.14
Linoleic acid	82.7 (63.6-108.7)	112.8 (76.2-148.5)	0.002	119.0 (92.7-162)	442 (229-536) *	0.001
Arachidonic acid	3.7 (2.7-5.4)	5.3 (3.7-6.2)	0.009	5.9 (4.6-6.3)	8.7 (7.8-9.1) *	0.001
%UFA	61.5 (60-64)	63.8 (62.5-66.3)	0.003	48.2 (40.1-67.6)	69.0 (59.3-73.6) *	<0.001
Unbound FA	3.45 (2.48-4.67)	4.75 (2.4-8.4)	0.04	5.4 (4.5-5.7)	19.4 (16.0-21.0)*	<0.001

UFA: unsaturated fatty acids, FA: fatty acid * Significantly different from control group.

<u>Table S5:</u> Clinical characteristics of 116 patients admitted to the ICU comparing hospital survivors versus non survivors. Related to table 2

Variable	Survivors (103)	Non-survivors (13)	P value
Age in years, median (IQR)	64 (50-75)	65 (51-75)	0.70
Female sex, n (%)	36 (35)	4 (31)	0.99
BMI in Kg/m^2, median (IQR)	28.7 (25.1-33.5)	31.4 (24.9-35.1)	0.45
Race, n (%)			0.51
Caucasian	70 (68)	10 (77)	
Hispanic	13 (12)	0	
Asian	4 (4)	0	
African American	2 (2)	0	
Native American	14 (14)	3 (23)	
Diabetes Mellitus, n (%)	38 (37)	8 (61)	0.13
Hypertension, n (%)	44 (43)	5 (38)	0.99
Coronary artery disease, n (%)	29 (28)	4 (31)	0.99
Cancer, n (%)	12 (12)	2 (15)	0.65
Cerebrovascular disease, n (%)	8 (8)	1 (8)	0.99
Cirrhosis, n (%)	4 (4)	3 (23)	0.03
Chronic kidney disease, n (%)	14 (14)	3 (23)	0.40
Immunosuppression, n (%)	12 (12)	1 (8)	0.99
Pre-ICU days, median (IQR)	0 (0-2)	1 (0-2)	0.53
ICU source, n (%)			0.04
Emergency Room	21 (21)	5 (38)	
Hospital Ward	26 (25)	6 (46)	
Operating Room	38 (37)	0 (0)	
Outside hospital	18 (17)	2 (15)	
Temperature in °Celsius, median (IQR)	37.5 (36.8-38.4)	36.8 (35.4-38.4)	0.26
Median arterial pressure in mmHg, median (IQR)	62 (58-70)	60 (56-70)	0.54
Heart rate, median (IQR)	97 (86-107)	111 (99-121)	0.009
Respiratory rate, median (IQR)	24 (20-29)	28 (20-31)	0.43
Fluids in first 24 hours, median (IQR)	2849 (1932-5275)	2438 (1612-4045)	0.47
Urine output, first 24 h, median (IQR)	1787 (1200-2425)	295 (30-2062)	0.02
Fluid balance 24 hours (mL), median (IQR)	1189 (-204-3299)	1554 (510-3280)	0.53
Glasgow Coma Scale, median (IQR)	15 (12-15)	14 (4-15)	0.07
Acute physiologic score, median (IQR)	42 (30-55)	75 (49-96)	0.002
APACHE IV, median (IQR)	55 (38-71)	96 (63-106)	0.002
Predicted mortality, median (IQR)	9.8 (2.4-23.5)	34 (21.2-62.8)	0.0004
Vasopressors on Day 1	60 (58)	9 (69)	0.55
SOFA score Day 1, median (IQR)	7 (5-9)	11 (4-13)	0.04
SOFA score Day 2, median (IQR)	5 (3-8)	11 (6-14)	0.0005
SOFA score Day 3, median (IQR)	5 (3-7)	10 (8-13)	0.0004
SOFA score Day 4, median (IQR)	4 (3-9)	9 (7-12)	0.006
SOFA score Day 5, median (IQR)	5 (3-10)	9 (7-13)	0.02
SOFA score Day 6, median (IQR)	5 (3-10)	9 (7-13)	0.04
SOFA score Day 7, median (IQR)	6 (4-10)	8 (8-12)	0.17
**Baseline laboratory results**			
White blood count ($x10^9$/L), median (IQR)	9.8 (6.9-14.3)	12 (8.9-17.6)	0.22
Platelets ($x10^9$/L), median (IQR)	171 (113-238)	165 (146-217)	0.73

Sodium (mmol/L), median (IQR)	138 (135-141)	135 (129-139)	0.04
Creatinine (mg/dL), median (IQR)	1.07 (0.79-1.66)	1.88 (1.02-3.02)	0.008
Blood urea nitrogen (mg/dL), median (IQR)	18 (12-26)	29 (17-48)	0.01
Glucose (mg/dL), median (IQR)	151 (119-181)	185 (107-291)	0.16
pH, median (IQR)	7.4 (7.35-7.43)	7.34 (7.21-7.4)	0.04
Lactate (mmol/L), median (IQR)	1.6 (1.1-2.8)	5.2 (1.6-8)	0.005
Bilirubin (mg/dL), median (IQR)	0.7 (0.4-1)	1 (0.3-2.7)	0.25
Hematocrit (%), median (IQR)	33 (28-38)	34 (27-37)	0.83
Lipase (U/L), median (IQR)	35 (15-68)	19 (12-80)	0.59
Albumin (g/dL), median (IQR)	3.5 (3.1-4)	3.3 (2.8-3.6)	0.11
Calcium (mg/dL), median (IQR)	8.2 (7.8-8.7)	8.2 (7.4-8.8)	0.81
Ionized Calcium (mg/dL), median (IQR)	4.5 (4.2-4.8)	4.2 (4-4.6)	0.06

IQR: interquartile range, n: number, BMI: body mass index, ICU: intensive care unit, APACHE: Acute Physiology and Chronic Health Evaluation, SOFA: Sequential Organ Failure Assessment.

Table S6: Fatty acid profile (in micromolars) of 116 ICU patients comparing hospital survivors versus non survivors, related to table 3

Fatty acids (μM)	Survivors (103)	Non-survivors (13)	P value
Myristic acid	7.4 (5.7-10.6)	9.2 (6.3-15.5)	0.11
Palmitic acid	137 (114-187)	192 (115-269)	0.11
Palmitoleic acid	15.8 (9.7-24.1)	20.1 (11.6-41.8)	0.16
Stearic acid	47.7 (37.2-55.3)	57.9 (46.5-79.9)	0.02
Oleic acid	225.9 (175-288)	358.9 (182.1-420.7)	0.05
Linoleic acid	86.6 (66.3-114.6)	121.4 (90.2-153.9)	0.03
Arachidonic acid	4.3 (3.3-5.5)	4.8 (3.7-7.1)	0.14
UFA	341.7 (260.2-450.6)	529.4 (292-605)	0.04
NEFA	547.5 (434.3-698.9)	787.3 (455.4-967.7)	0.04
SFA	193.1 (160.5-251.2)	260.7 (163.4-366.1)	0.09
Unbound FA	3.8 (2.8-5.9)	6.2 (4.5-11.8)	0.02

UFA: unsaturated fatty acid, NEFA: non-esterified fatty acid, SFA: saturated fatty acid, FA: fatty acid

Table S7: Outcomes and interventions of 116 patients comparing hospital survivors versus non survivors., related to table 2

Variable	Survivors (103)	Non-survivors (13)	P value
Multiorgan failure, n (%)	43 (42)	12 (92)	0.0006
Renal Replacement Therapy, n (%)	12 (12)	5 (38)	0.02
Mechanical ventilation, n (%)	58 (56)	6 (46)	0.56
Veno-venous ECMO, n (%)	8 (8)	4 (31)	0.03
Mechanical ventilation days, median (IQR)	1 (0.3-9)	29.3 (8.1-54.8)	0.003
ICU length of stay days, median (IQR)	3.1 (1.9-8.8)	4.9 (2.5-37.4)	0.06
Hospital length of stay days, median (IQR)	9.6 (6-21)	10.8 (3.6-38.5)	0.94
DVT or PE events, n (%)	12 (12)	1 (8)	0.99

ICU: intensive care unit, IQR: interquartile range, ECMO: extracorporeal membrane oxygenation, DVT: deep venous thrombosis, PE: pulmonary embolism

Table S8: Cytokine profile of 116 ICU patients comparing hospital survivors versus non survivors, related to table 6

Variable	Survivors (103)	Non-survivors (13)	P value
GROα (pg/ml)	26.3 (8.8-42.2)	33.3 (18.5-68.6)	0.1
IL-1β (pg/ml)	6.4 (0-16.5)	11 (4.4-22.5)	0.08
IL-1Rα (pg/ml)	9.7 (4.1-44.5)	160.3 (24.2-394.8)	0.0002
IL-4 (pg/ml)	2.8 (0.4-7.2)	0.4 (0-5.6)	0.11
IL-6 (pg/ml)	181.8 (50.6-453.6)	439.7 (131.1-854.4)	0.05
IL-18 (pg/ml)	102 (38.2-150.6)	119.4 (53.4-216.7)	0.51
IP-10 (pg/ml)	421.5 (149-2834.4)	1683.6 (212.4-3877.7)	0.24
MCP-1 (pg/ml)	740.9 (447.2-1336.4)	1150.2 (519.6-1638.9)	0.25

<u>Table S9:</u> Calcium and Albumin levels in 3969 hospitalized COVID-19 patients comparing patients that survived versus those that did not survive hospitalization, related to table 4

Variable	Survivors (3398)		Non-survivors (571)		p-value
	Mean	STD DEV	Mean	STD DEV	
Calcium (mg/dL) admission	**8.48**	**0.69**	**8.26**	**0.87**	**0.001**
Calcium day 1	**8.21**	**0.63**	**7.82**	**0.80**	**0.001**
Calcium day 2	**8.23**	**0.65**	**7.73**	**0.83**	**0.001**
Calcium day 3	**8.23**	**0.63**	**7.87**	**0.75**	**0.001**
Calcium day 4	**8.26**	**0.63**	**7.91**	**0.76**	**0.001**
Albumin (g/dL) admission	**3.36**	**0.56**	**3.08**	**0.56**	**0.001**
Albumin day 1	**3.00**	**0.56**	**2.74**	**0.50**	**0.001**
Albumin day 2	**2.93**	**0.55**	**2.62**	**0.50**	**0.001**
Albumin day 3	**2.82**	**0.55**	**2.58**	**0.50**	**0.001**
Albumin day 4	**2.77**	**0.55**	**2.50**	**0.47**	**0.001**

STD: standard deviation.

Table S10: Univariate analysis evaluating the association of multiple variables and hospital mortality in 3969 hospitalized COVID-19 patients. Related to table 5.

Variable	OR	CI 95%		p value
		CI lower	CI upper	
Calcium (mg/dL) admission	0.646	0.567	0.737	0.001
Calcium day 1	0.397	0.328	0.48	0.001
Calcium day 2	0.326	0.265	0.4	0.001
Calcium day 3	0.432	0.354	0.528	0.001
Calcium day 4	0.454	0.355	0.58	0.001
Alb (g/dL) admission	0.424	0.357	0.504	0.001
Albumin day 1	0.423	0.332	0.54	0.001
Albumin day 2	0.348	0.268	0.452	0.001
Albumin day 3	0.442	0.338	0.577	0.001
Albumin day 4	0.385	0.285	0.52	0.001
Age (years)	1.042	1.036	1.048	0.001
Male sex	1.343	1.124	1.606	0.001
HTN	1.53	1.276	1.834	0.001
DM	1.285	1.068	1.546	0.008
CAD	1.725	1.353	2.2	0.001
CKD	1.8	1.339	2.418	0.001
CHF	1.95	1.473	2.582	0.001
h/o Malignancy	1.662	1.252	2.207	0.001
BMI	0.985	0.975	0.996	0.008
Immunosuppression	0.783	0.354	1.732	0.546
CRP (mg/dL) admission	1.001	1	1.002	0.109
Creatinine (mg/dL) admission	1.033	1.002	1.064	0.035
BUN (mg/dL) admission	1.018	1.015	1.021	0.001
WBC ($x10^9$/L) admission	1.004	0.983	1.026	0.706
PLT ($x10^9$/L) admission	0.999	0.998	1	0.123

OR: odds ratio, CI: Confidence Interval, BUN: blood urea nitrogen; CAD: coronary artery disease; CKD: chronic kidney disease; CHF: congestive heart failure; BMI: body mass index; CRP: C-reactive protein; BUN: blood urea nitrogen; WBC: white blood count; PLT: platelets.

Table S11: Multivariate analysis evaluating the association of hospital mortality with serum levels of calcium on admission, related to table 5.

Variable	OR	95% C.I.		P value
		Lower	Upper	
Age	1.04	1.032	0.001	0.001
Male	1.442	1.177	0.001	0.001
HTN	1.088	0.865	0.471	0.471
DM	1.05	0.839	0.669	0.669
CAD	1.015	0.763	0.92	0.92
CKD	1.246	0.868	0.234	0.234
CHF	1.171	0.842	0.347	0.347
Malignancy	1.038	0.743	0.827	0.827
BMI	1.028	1.016	0.001	0.001
Creatinine admission	0.965	0.913	0.218	0.218
BUN admission	1.014	1.009	0.001	0.001
Calcium admission	1.002	0.957	0.948	0.948

OR: odds ratio, CI: Confidence Interval, BUN: blood urea nitrogen; HTN: hypertension, DM: diabetes mellitus, CAD: coronary artery disease; CKD: chronic kidney disease; CHF: congestive heart failure; BMI: body mass index; BUN: blood urea nitrogen.

Table S12: Multivariate analysis evaluating the association of hospital mortality with serum levels of calcium on day 1. Related to table 5.

Variable	OR	95% C.I.		P value
		Lower	Upper	
Age	1.033	1.023	1.043	0.001
Male	1.291	0.996	1.672	0.053
HTN	1.103	0.825	1.475	0.509
DM	1.1	0.828	1.459	0.511
CAD	0.812	0.553	1.19	0.285
CKD	1.189	0.751	1.883	0.46
CHF	1.385	0.908	2.113	0.131
Malignancy	1.012	0.642	1.596	0.957
BMI	1.025	1.01	1.04	0.001
Creatinine admission	0.958	0.895	1.025	0.209
BUN admission	1.012	1.006	1.018	0.001
Calcium day 1	0.438	0.357	0.539	0.001

OR: odds ratio, CI: Confidence Interval, BUN: blood urea nitrogen; HTN: hypertension, DM: diabetes mellitus, CAD: coronary artery disease; CKD: chronic kidney disease; CHF: congestive heart failure; BMI: body mass index; BUN: blood urea nitrogen.

Table S13: Multivariate analysis evaluating the association of hospital mortality with serum levels of calcium on day 2. Related to table 5.

Variable	OR	95% C.I.		P value
		Lower	Upper	
Age	1.027	1.016	1.038	0.001
Male	1.518	1.149	2.005	0.003
HTN	1.128	0.823	1.544	0.454
DM	1.082	0.804	1.457	0.602
CAD	0.862	0.585	1.268	0.45
CKD	1.378	0.871	2.183	0.171
CHF	1.364	0.91	2.044	0.133
Malignancy	1.007	0.627	1.619	0.976
BMI	1.033	1.018	1.049	0.001
Creatinine admission	0.953	0.884	1.028	0.212
BUN admission	1.008	1.001	1.014	0.018
Calcium day 2	0.348	0.279	0.435	0.001

OR: odds ratio, CI: Confidence Interval, BUN: blood urea nitrogen; HTN: hypertension, DM: diabetes mellitus, CAD: coronary artery disease; CKD: chronic kidney disease; CHF: congestive heart failure; BMI: body mass index; BUN: blood urea nitrogen.

Table S14: Multivariate analysis evaluating the association of hospital mortality with serum levels of calcium on day 3. Related to table 5.

Variable	OR	95% C.I.		P value
		Lower	Upper	
Age	1.031	1.02	1.042	0.001
Male	1.435	1.083	1.901	0.012
HTN	1.33	0.965	1.832	0.081
DM	0.987	0.729	1.336	0.93
CAD	0.825	0.551	1.235	0.349
CKD	1.162	0.702	1.924	0.559
CHF	1.439	0.944	2.192	0.09
Malignancy	0.784	0.479	1.286	0.336
BMI	1.037	1.02	1.053	0.001
Creatinine admission	0.978	0.896	1.069	0.628
BUN admission	1.005	0.998	1.012	0.157
Calcium day 3	0.457	0.367	0.568	0.001

OR: odds ratio, CI: Confidence Interval, BUN: blood urea nitrogen; HTN: hypertension, DM: diabetes mellitus, CAD: coronary artery disease; CKD: chronic kidney disease; CHF: congestive heart failure; BMI: body mass index; BUN: blood urea nitrogen.

Table S15: Multivariate analysis evaluating the association of hospital mortality with serum levels of calcium on day 4. Related to table 5.

Variable	OR	95% C.I.		P value
		Lower	Upper	
Age	1.023	1.009	1.037	0.001
Male	1.852	1.279	2.684	0.001
HTN	1.48	0.975	2.245	0.065
DM	0.975	0.666	1.427	0.895
CAD	0.829	0.487	1.41	0.489
CKD	1.446	0.789	2.652	0.233
CHF	1.176	0.656	2.109	0.586
Malignancy	1.042	0.527	2.059	0.906
BMI	1.02	1	1.04	0.048
Creatinine admission	0.926	0.839	1.022	0.125
BUN admission	1.009	1.001	1.017	0.032
Calcium day 4	0.498	0.382	0.649	0.001

OR: odds ratio, CI: Confidence Interval, BUN: blood urea nitrogen; HTN: hypertension, DM: diabetes mellitus, CAD: coronary artery disease; CKD: chronic kidney disease; CHF: congestive heart failure; BMI: body mass index; BUN: blood urea nitrogen.

Table S16: Multivariate analysis evaluating the association of hospital mortality with serum albumin on admission. Related to table 5.

Variable	OR	95% C.I.		P value
		Lower	Upper	
Age	1.037	1.028	1.046	0.001
Male	1.408	1.131	1.754	0.002
HTN	1.079	0.843	1.381	0.546
DM	0.932	0.733	1.185	0.564
CAD	1.022	0.75	1.391	0.892
CKD	1.11	0.753	1.637	0.598
CHF	1.084	0.764	1.538	0.652
Malignancy	1.132	0.789	1.624	0.5
BMI	1.03	1.017	1.043	0.001
Creatinine admission	0.989	0.933	1.049	0.714
BUN admission	1.007	1.002	1.013	0.006
Albumin on admission	0.442	0.364	0.537	0.001

OR: odds ratio, CI: Confidence Interval, BUN: blood urea nitrogen; HTN: hypertension, DM: diabetes mellitus, CAD: coronary artery disease; CKD: chronic kidney disease; CHF: congestive heart failure; BMI: body mass index; BUN: blood urea nitrogen.

Table S17: Multivariate analysis evaluating the association of hospital mortality with serum albumin on day 1. Related to table 5.

Variable	OR	95% C.I.		P value
		Lower	Upper	
Age	1.036	1.025	1.048	0.001
Male	1.383	1.021	1.875	0.036
HTN	0.957	0.68	1.346	0.799
DM	0.998	0.717	1.388	0.988
CAD	0.962	0.622	1.486	0.86
CKD	0.805	0.461	1.407	0.447
CHF	1.262	0.769	2.071	0.358
Malignancy	1.403	0.831	2.371	0.205
BMI	1.045	1.028	1.063	0.001
Creatinine admission	1.074	0.986	1.17	0.101
BUN admission	1.002	0.995	1.009	0.6
Albumin on day 1	0.401	0.302	0.532	0.001

OR: odds ratio, CI: Confidence Interval, BUN: blood urea nitrogen; HTN: hypertension, DM: diabetes mellitus, CAD: coronary artery disease; CKD: chronic kidney disease; CHF: congestive heart failure; BMI: body mass index; BUN: blood urea nitrogen.

Table S18: Multivariate analysis evaluating the association of hospital mortality with serum albumin on day 2. Related to table 5.

Variable	OR	95% C.I.		P value
		Lower	Upper	
Age	1.041	1.028	1.054	0.001
Male	1.651	1.202	2.269	0.002
HTN	1.18	0.825	1.689	0.365
DM	0.902	0.644	1.263	0.548
CAD	0.885	0.559	1.401	0.601
CKD	1.24	0.724	2.125	0.433
CHF	1.172	0.73	1.881	0.512
Malignancy	1.047	0.606	1.809	0.87
BMI	1.044	1.026	1.063	0.001
Creatinine admission	1.012	0.93	1.101	0.782
BUN admission	1.001	0.993	1.008	0.884
Albumin on day 2	0.293	0.216	0.397	0.001

OR: odds ratio, CI: Confidence Interval, BUN: blood urea nitrogen; HTN: hypertension, DM: diabetes mellitus, CAD: coronary artery disease; CKD: chronic kidney disease; CHF: congestive heart failure; BMI: body mass index; BUN: blood urea nitrogen.

Table S19: Multivariate analysis evaluating association of hospital mortality with serum levels of albumin on day 3. Related to table 5.

Variable	OR	95% C.I.		P value
		Lower	Upper	
Age	1.042	1.029	1.055	0.001
Male	1.458	1.052	2.02	0.023
HTN	1.154	0.802	1.661	0.44
DM	0.854	0.602	1.211	0.375
CAD	1.024	0.642	1.633	0.922
CKD	0.767	0.431	1.364	0.367
CHF	0.865	0.503	1.487	0.6
Malignancy	1.063	0.612	1.848	0.828
BMI	1.044	1.026	1.062	0.001
Creatinine admission	1.069	0.977	1.169	0.145
BUN admission	1.003	0.995	1.011	0.512
Albumin on day 3	0.366	0.268	0.501	0.001

OR: odds ratio, CI: Confidence Interval, BUN: blood urea nitrogen; HTN: hypertension, DM: diabetes mellitus, CAD: coronary artery disease; CKD: chronic kidney disease; CHF: congestive heart failure; BMI: body mass index; BUN: blood urea nitrogen.

Table S20: Multivariate analysis evaluating association of hospital mortality with serum levels of albumin on day 4. Related to table 5.

Variable	OR	95% C.I.		P value
		Lower	Upper	
Age	1.039	1.025	1.053	0.001
Male	1.457	1.021	2.078	0.038
HTN	1.148	0.768	1.717	0.5
DM	0.779	0.535	1.134	0.193
CAD	1.091	0.651	1.83	0.74
CKD	1.205	0.639	2.272	0.564
CHF	0.737	0.4	1.357	0.327
Malignancy	0.84	0.435	1.62	0.602
BMI	1.035	1.016	1.054	0.001
Creatinine admission	1.02	0.922	1.129	0.699
BUN admission	0.999	0.99	1.008	0.838
Albumin on day 3	0.327	0.232	0.46	0.001

OR: odds ratio, CI: Confidence Interval, BUN: blood urea nitrogen; HTN: hypertension, DM: diabetes mellitus, CAD: coronary artery disease; CKD: chronic kidney disease; CHF: congestive heart failure; BMI: body mass index; BUN: blood urea nitrogen.

TABLE S21: Cytokine profile of ICU patients comparing COVID versus non-COVID patients (left side) as well as animal experiment comparing control mice versus mice treated with linoleic acid (right side), related to table 6.

Variable	Non-COVID, Non-Septic shock (49)	COVID-19 (39)	P value	Control mice	Linoleic acid mice	P value
GROα (pg/ml)	12.6 (5.1-27.1)	35 (18.6-53.8)	<0.001	685 (480-1018)	65034 (63915-67163)	0.001
IL-1β (pg/ml)	0 (0-5.1)	8.3 (4.6-16.5)	<0.001	8.5 (1.2-19.0)	26.8 (15.8-46.8)	0.01
IL-1Rα (pg/ml)	4.8 (2.6-12.9)	22.4 (9.1-53.1)	<0.001			
IL-4 (pg/ml)	2.3 (0-6.4)	4.7 (1.4-6.9)	0.03	5.0 (0.6-8.1)	7.5 (5.6-7.5)	0.34
IL-6 (pg/ml)	143.8 (33.2-357.1)	360 (67-641)	0.006	22.8 (3.1-155)	5828 (4383-11758)	0.001
IL-18 (pg/ml)	32.8 (19.6-47.4)	119.4 (86.4-174.2)	<0.001			
IP-10 (pg/ml)	175 (103-285)	3204 (1956-3941)	< 0.001	576 (446-783)	926 (573-1318)	0.23
MCP-1 (pg/ml)	561 (397-752)	1336 (892-1836)	< 0.001	126 (59-339)	2350 (1993-2526)	0.001
TNFα (pg/ml)	13 (5.5-23.4)	40.4 (28.3-58.4)	< 0.001	25.0 (13.7-43.5)	120 (108-127)	0.001

<u>TABLE S22:</u> Outcomes and interventions of 88 patients admitted to the ICU comparing COVID versus non-COVID, non-septic patients, related to table 7.

Variable	Non-COVID, Non-Septic shock (49)	COVID-19 (39)	P value
Multiorgan failure, n (%)	17 (35)	23 (59)	0.02
Renal Replacement Therapy, n (%)	3 (6)	9 (23)	0.02
Veno-venous ECMO, n (%)	1 (2)	10 (26)	0.001
ICU Mortality, n (%)	2 (4)	5 (12.8)	0.23
Mechanical ventilation days, median (IQR)	0.35 (0.24-0.60)	25.8 (9.4-44.9)	< 0.0001
ICU length of stay days, median (IQR)	2.1 (1-3.2)	14.5 (5.6-34.6)	< 0.0001
Hospital length of stay days, median (IQR)	6.9 (5.1-12.7)	22.9 (10.7-42.2)	< 0.0001
28 Day Mortality, n (%)	2 (4)	5 (12.8)	0.23
DVT or PE events	1 (2)	10 (26)	0.002

ICU: intensive care unit, IQR: interquartile range, ECMO: extracorporeal membrane oxygenation, DVT: deep venous thrombosis, PE: pulmonary embolism

TABLE S23: Clinical characteristics, fatty acid profile, cytokine profile, and outcomes of 70 patients without diabetes admitted to the ICU comparing COVID versus non-COVID patients, related to tables 2, 3, 6 and 7.

Variable	Non-COVID (50)	COVID-19 (20)	P value
Age in years, median (IQR)	67 (54-75)	49 (42-59)	0.0002
Female sex, n (%)	16 (32)	8 (40)	0.58
BMI in Kg/m^2, median (IQR)	27.3 (23.4-31.8)	31.8 (27.6-39.3)	0.003
Caucasians, n (%)	45 (90)	5 (25)	<0.0001
Hypertension, n (%)	19 (38)	3 (15)	0.08
Coronary artery disease, n (%)	16 (32)	0 (0)	0.003
Cancer, n (%)	6 (12)	2 (10)	0.99
Cerebrovascular disease, n (%)	9 (18)	0 (0)	0.052
Cirrhosis, n (%)	4 (8)	0 (0)	0.32
Chronic kidney disease, n (%)	6 (12)	1 (5)	0.66
Immunosuppression, n (%)	5 (10)	1 (5)	0.66
Mechanical Ventilation, n (%)	30 (60)	12 (60)	0.99
Acute physiologic score, median (IQR)	42.5 (31-55.2)	35 (22-55.2)	0.21
APACHE IV, median (IQR)	56 (39-74)	40 (32-64)	0.03
Predicted mortality, median (IQR)	6.6 (1.5-25.8)	10.6 (5.8-26.8)	0.14
Vasopressors on Day 1	31 (62)	10 (50)	0.42
Lipase (U/L), median (IQR)	22 (11-43)	87 (48-144)	<0.0001
Albumin (g/dL), median (IQR)	4 (3.2-4.1)	3.2 (2.9-3.4)	0.001
Calcium (mg/dL), median (IQR)	8.4 (7.8-9)	8 (7.5-8.2)	0.01
Ionized Calcium (mg/dL), median (IQR)	4.6 (4.3-5)	4.3 (4.1-4.6)	0.01
**FATTY ACID PROFILE**			
Myristic acid (μM)(IQR)	8.4 (6.3-10.7)	6.8 (5.5-11.1)	0.64
Palmitic acid(μM)(IQR)	135.3 (113.3-178.2)	189 (115-238.4)	0.04
Palmitoleic acid (μM)(IQR)	16 (11-23)	19 (12-27)	0.3
Stearic acid (μM)(IQR)	48 (38-55)	49 (38-59)	0.67
Oleic acid(μM)(IQR)	228 (174-273)	277 (193-371)	0.08
Linoleic acid(μM)(IQR)	84 (58-108)	134 (85-171)	0.0006
Arachidonic acid(μM)(IQR)	3.6 (3-5.2)	5 (3.6-5.7)	0.009
NEFA(μM)(IQR)	539 (398-640)	689 (471-920)	0.05
UFA(μM)(IQR)	341 (250-403)	461 (320-600)	0.03
SFA(μM)(IQR)	189 (166-241)	251 (162-331)	0.11
%UFA	62.5 (60.2-64.4)	64 (62.5-66)	0.03
Unbound FA (μM)(IQR)	3.48 (2.72-5.2)	5.2 (2.32-9.12)	0.13
**CYTOKINES**			
GROα (pg/ml)	19.2 (4.9-33.2)	38.6 (20.7-62.3)	0.007
IL-1β (pg/ml)	2.7 (0-14.2)	9.2 (3.7-16)	0.06
IL-1Rα (pg/ml)	7.8 (2.8-32.5)	32.9 (11.2-52.6)	0.004
IL-4 (pg/ml)	2.5 (0-6.3)	4.6 (1.8-6.9)	0.13
IL-6 (pg/ml)	125 (32-422)	426 (90-1125)	0.01
IL-18 (pg/ml)	53.4 (23.6-153.2)	135.7 (77.6-176-7)	0.17
IP-10 (pg/ml)	228 (119-564)	3322 (2095-4060)	<0.0001
MCP-1 (pg/ml)	560 (381-1034)	1324 (1051-2094)	0.0002
TNFα (pg/ml)	16 (6-38.5)	39.4 (27.9-70.4)	0.002

OUTCOMES			
Multiorgan failure, n (%)	19 (38)	11 (60)	0.11
Renal Replacement Therapy, n (%)	3 (6)	4 (20)	0.09
Veno-venous ECMO, n (%)	1 (2)	5 (25)	0.007
ICU Mortality, n (%)	3 (6)	2 (10)	0.62
Mechanical ventilation days, median (IQR)	0.4 (0.2-3.3)	20 (9-56)	<0.0001
ICU length of stay days, median (IQR)	2.2 (1.1-4.1)	13.2 (3.3-44.7)	<0.0001
Hospital length of stay days, median (IQR)	7.4 (5.1-14.9)	20.7 (10.6-54.3)	0.0004
28 Day Mortality, n (%)	3 (6)	2 (10)	0.61
DVT or PE events, n (%)	3 (6)	5 (25)	0.03

TABLE S24: Clinical characteristics, fatty acid profile, cytokine profile, and outcomes of 76 male patients admitted to the ICU comparing COVID versus non-COVID patients, related to tables 2, 3, 6 and 7.

Variable	Non-COVID (54)	COVID-19 (22)	P value
Age in years, median (IQR)	71 (61-77)	54 (44-63)	<0.0001
BMI in Kg/m^2, median (IQR)	27.5 (25-32.5)	28.8 (26.3-33.7)	0.31
Caucasians, n (%)	49 (91)	6 (27)	<0.0001
Diabetes Mellitus, n (%)	20 (37)	10 (45)	0.61
Hypertension, n (%)	25 (46)	6(27)	0.19
Coronary artery disease, n (%)	23 (43)	3 (14)	0.01
Cancer, n (%)	10 (19)	1 (5)	0.16
Cerebrovascular disease, n (%)	3 (6)	2 (9)	0.62
Cirrhosis, n (%)	4 (7)	0(0)	0.31
Chronic kidney disease, n (%)	9 (17)	2 (9)	0.49
Immunosuppression, n (%)	4 (7)	2 (9)	0.99
Acute physiologic score, median (IQR)	46.5 (33-61)	37.5 (22-57)	0.11
APACHE IV, median (IQR)	61 (47-83)	47 (29-66)	0.01
Predicted mortality, median (IQR)	2.3 (1.3-25.2)	11.7 (6.2-26)	0.1
Vasopressors on Day 1	39 (72)	9 (41)	0.01
Lipase (U/L), median (IQR)	20 (9-35)	73 (42-124)	<0.0001
Albumin (g/dL), median (IQR)	4 (3.2-4.1)	3.2 (2.6-3.4)	0.0003
Calcium (mg/dL), median (IQR)	8.3 (7.9-9)	7.8 (7.4-8.2)	0.001
Ionized Calcium (mg/dL), median (IQR)	4.5 (4.2-4.8)	4.4 (4.3-4.7)	0.26
**FATTY ACID PROFILE**			
Myristic acid (μM)(IQR)	7.9 (5.6-11.3)	6.6 (4.8-9.2)	0.09
Palmitic acid(μM)(IQR)	135 (113-180)	164 (111-217)	0.25
Palmitoleic acid (μM)(IQR)	13.7 (8.6-22.9)	15.1 (7.1-20.7)	0.87
Stearic acid (μM)(IQR)	50.3 (39.6-56.7)	48.4 (43.1-57.4)	0.99
Oleic acid(μM)(IQR)	220 (159-275)	232 (176-316)	0.48
Linoleic acid(μM)(IQR)	82.3 (60.8-109.7)	116.5 (79.8-146.7)	0.01
Arachidonic acid(μM)(IQR)	3.9 (3-4.8)	5.3 (3.6-6.2)	0.009
NEFA(μM)(IQR)	535 (397-674)	578 (438-811)	0.31
UFA(μM)(IQR)	333 (241-413)	360 (265-514)	0.25
SFA(μM)(IQR)	192 (163-245)	223 (160-277)	0.39
%UFA	61.5 (60-64)	62.7 (60-66)	0.22
Unbound FA (μM)(IQR)	3.5 (2.8-5.4)	4.7 (2.1-8.1)	0.32
**CYTOKINES**			
GROα (pg/ml)	17 (6.8-29.6)	25.5 (15.7-47.3)	0.04
IL-1β (pg/ml)	2.7 (0-17.2)	10.6 (5.5-18.4)	0.04
IL-1Rα (pg/ml)	8.6 (3.7-116)	21.6 (9.2-43.4)	0.11
IL-4 (pg/ml)	3 (0-6.4)	4.7 (1.7-7.9)	0.14
IL-6 (pg/ml)	172 (71-430)	358 (147-716)	0.01
IL-18 (pg/ml)	49 (23.6-107.4)	140 (98.4-187)	0.009
IP-10 (pg/ml)	198 (120-380)	3577 (1515-4074)	<0.0001
MCP-1 (pg/ml)	645 (415-913)	1473 (989-2579)	<0.0001
TNFα (pg/ml)	21.2 (6.5-61)	41 (28.3-76.7)	0.009

OUTCOMES			
Multiorgan failure, n (%)	22 (41)	13 (59)	0.2
Renal Replacement Therapy, n (%)	6 (11)	4 (18)	0.46
Veno-venous ECMO, n (%)	1 (2)	3 (14)	0.04
ICU Mortality, n (%)	5 (9)	3 (14)	0.68
Mechanical ventilation days, median (IQR)	0.4 (0.2-0.8)	20.3 (8.9-38.8)	<0.0001
ICU length of stay days, median (IQR)	2.1 (1.2-3.5)	14.2 (4.6-27.7)	<0.0001
Hospital length of stay days, median (IQR)	7 (5.1-12)	22.8 (11.3-34.5)	<0.0001
28 Day Mortality, n (%)	6 (11)	3 (14)	0.71
DVT or PE events, n (%)	1 (2)	7 (32)	0.0005
